# Connection between protein-tyrosine kinase inhibition and coping with oxidative stress in *Bacillus subtilis*

**DOI:** 10.1073/pnas.2321890121

**Published:** 2024-06-10

**Authors:** Lei Shi, Abderahmane Derouiche, Santosh Pandit, Meshari Alazmi, Magali Ventroux, Julie Bonne Køhler, Marie-Francoise Noirot-Gros, Xin Gao, Ivan Mijakovic

**Affiliations:** ^a^Systems and Synthetic Biology Division, Department of Biology and Biological Engineering, Chalmers University of Technology, Gothenburg SE-412 96, Sweden; ^b^Computational Bioscience Research Center Computer, Electrical and Mathematical Sciences and Engineering Division, King Abdullah University of Science and Technology, Thuwal 23955-6900, Kingdom of Saudi Arabia; ^c^Department of Artificial Intelligence, College of Computer Science and Engineering, University of Ha'il, HailHa’il 81411, Saudi Arabia; ^d^Computer Science Program Computer, Electrical and Mathematical Sciences and Engineering Division, King Abdullah University of Science and Technology, Thuwal 23955-6900, Kingdom of Saudi Arabia; ^e^Micalis Institute, INRAE, AgroParisTech, Université Paris-Saclay, Jouy-en-Josas 78352, France; ^f^Technical University of Denmark (DTU) Biosustain, The Novo Nordisk Foundation Center for Biosustainability, Technical University of Denmark, Lyngby DK-2800, Denmark

**Keywords:** protein-tyrosine phosphorylation, oxidative stress, bacterial protein-tyrosine kinases

## Abstract

In bacteria, attenuation of protein-tyrosine phosphorylation occurs during oxidative stress. The main described mechanism behind this effect is the H_2_O_2_-triggered conversion of bacterial phospho-tyrosines to protein-bound 3,4-dihydroxyphenylalanine. This disrupts the bacterial tyrosine phosphorylation-based signaling network, which alters the bacterial polysaccharide biosynthesis. Herein, we report an alternative mechanism, in which oxidative stress leads to a direct inhibition of bacterial protein-tyrosine kinases (BY-kinases). We show that DefA, a minor peptide deformylase, inhibits the activity of BY-kinase PtkA when *Bacillus subtilis* is exposed to oxidative stress. High levels of PtkA activity are known to destabilize *B. subtilis* pellicle formation, which leads to higher sensitivity to oxidative stress. Interaction with DefA inhibits both PtkA autophosphorylation and phosphorylation of its substrate Ugd, which is involved in exopolysaccharide formation. Inactivation of *defA* drastically reduces the capacity of *B. subtilis* to cope with oxidative stress, but it does not affect the major oxidative stress regulons PerR, OhrR, and Spx, indicating that PtkA inhibition is the main pathway for DefA involvement in this stress response. Structural analysis identified DefA residues Asn95, Tyr150, and Glu152 as essential for interaction with PtkA. Inhibition of PtkA depends also on the presence of a C-terminal α-helix of DefA, which resembles PtkA-interacting motifs from known PtkA activators, TkmA, SalA, and MinD. Loss of either the key interacting residues or the inhibitory helix of DefA abolishes inhibition of PtkA in vitro and impairs postoxidative stress recovery in vivo, confirming the involvement of these structural features in the proposed mechanism.

## Significance

Protein-tyrosine phosphorylation is a widespread posttranslational modification. It is involved in the regulation of important cellular processes including cell growth, differentiation, death, pathogenicity, and response to oxidative stress. Eukaryotic organisms usually encounter a burst of tyrosine phosphorylation in response to oxidative stress due to the inactivation of tyrosine phosphatases by reactive oxygen species (ROS). In bacteria, tyrosine phosphorylation levels drop during oxidative stress due to the conversion of phospho-tyrosines to 3,4-dihydroxyphenylalanine, triggered by ROS. We report another mechanism of attenuating bacterial tyrosine phosphorylation during oxidative stress. A minor peptide deformylase DefA changes its structure during oxidative stress, interacts with a bacterial protein-tyrosine kinase PtkA, inhibits it, which leads to exopolysaccharide remodeling and helps bacteria cope with oxidative stress.

Protein-tyrosine phosphorylation is recognized as one of the main players in coping with redox stress. In mammalian cells, H_2_O_2_ stress is commonly associated with an increase in tyrosine phosphorylation, which is typically caused by the inactivation of a key cysteine residue in the active site of protein-tyrosine phosphatases (1). By contrast, in bacteria, tyrosine phosphorylation is usually attenuated during H_2_O_2_ stress. Bacterial protein-tyrosine phosphorylation is catalyzed by a unique class of protein-tyrosine kinases, the so-called bacterial tyrosine kinases (BY-kinases) (2). Interestingly, the main reported mechanism for the decrease of tyrosine phosphorylation upon H_2_O_2_ stress does not involve inhibition of BY-kinases. The reduction of tyrosine phosphorylation in intestinal and pulmonary pathogens, including *Listeria monocytogenes*, *Salmonella enterica* serovar Typhimurium, and *Klebsiella pneumoniae* (3), is reported to be caused by the conversion of phospho-tyrosines to protein-bound 3,4-dihydroxyphenylalanine, triggered by H_2_O_2_. This disrupts the bacterial tyrosine phosphorylation-based signaling networks, which alters the bacterial polysaccharide biosynthesis and weakens bacterial fitness during invasion. There is a single case in the literature where a direct inhibition of an atypical extracellular BY-kinase in *Campylobacter jejuni* is caused by reactive oxygen species (4). As far as we were able to ascertain, oxidative stress is not recognized as a causative agent in inhibition of classical cytosolic BY-kinases during oxidative stress.

BY-kinases share similarity with ATPases, which possess Walker A, Walker A′, and Walker B ATP-binding motifs. In addition, BY-kinases also possess a C-terminal tyrosine cluster, containing autophosphorylation sites (5). In gram-negative bacteria, BY-kinases are found in the form of a single polypeptide, containing a N-terminal transmembrane activator domain and a C-terminal BY-kinase catalytic domain. In gram-positive bacteria, BY-kinases are present as two separate proteins: a transmembrane activator protein and a cytoplasmic protein bearing the BY-kinase catalytic site. The interaction of the activator domain/protein with the catalytic domain of BY-kinases activates the kinase by stabilizing the ATP-binding pocket (6). Bacterial protein-tyrosine phosphorylation mediated by BY-kinases is involved in numerous biological processes, including extracellular polysaccharide (EPS) synthesis, biofilm formation, DNA replication, DNA damage repair, metabolic regulation, heat-shock response, and virulence (7). One of the most extensively characterized BY-kinase is the PtkA in *Bacillus subtilis* (8). Its principal protein substrate is a UDP-glucose dehydrogenase, Ugd. PtkA-dependent phosphorylation of Ugd increases its enzymatic activity, resulting in faster conversion of UDP-glucose to UDP-glucuronate, which is a substrate for synthesis of teichuronic acid, a specific type of alternative EPS in *B. subtilis*. Besides Ugd, other substrates of PtkA have been identified, including the single-stranded DNA binding protein SsbA, single-stranded DNA exonuclease YorK, aspartate semialdehyde dehydrogenase Asd, and class I heat-shock protein DnaK (7, 9). PtkA was found to not only affect the activity of its substrates but in some cases also govern their subcellular localization by phosphorylation (10).

Precise levels of BY-kinase activity (corresponding to precise levels of autophosphorylation) and cycles of phosphorylation/dephosphorylation are known to control biofilm formation and EPS synthesis. In *Escherichia coli*, the BY-kinase Wzc and its cognate tyrosine phosphatase Wzb are jointly responsible for group 1 capsule and colanic acid polysaccharide biosynthesis (11). The export of EPS is driven by octameric Wzc. Wzc autophosphorylation causes octamer dissociation and inactivation. The reactivation depends on the dephosphorylation of Wzc by Wzb. Similarly, in *Porphyromonas gingivalis*, the deletion of protein tyrosine kinase Ptk1 resulted in loss of EPS secretion, and the deletion of tyrosine phosphatase Php1 led to less EPS production (12). In *Streptococcus pneumoniae*, no detectable mature capsule was observed in the deletion strain of either BY-kinase CpsD or its activator CpsC (13, 14). The phosphorylation/dephosphorylation balance is also very important for biofilm formation in *B. subtilis*. Loss of either PtkA or PtpZ is known to impair *B. subtilis* biofilm development (15). It has been shown that EPS can protect bacterial cells from oxidative damage. In *Gluconacetobacter diazotrophicus*, an EPS-defective mutant was hypersensitive to H_2_O_2,_ and addition of purified EPS rescued the deficiency (16). In *Myxococcus xanthus*, the overall presence and integrity of an EPS layer plays a key role in the tolerance to harmful compounds, including oxidative stress-inducing chemicals (17). EPS synthesis is usually related to biofilm formation, exemplified by enhanced biofilm formation of *Acinetobacter oleivorans* in response to exposure to H_2_O_2_ (18).

In our previous genomic-wide yeast two-hybrid study aimed at detecting new substrates and activators of *B*. *subtilis* BY-kinase PtkA (19), DefA, a minor peptide deformylase (PDF), was found to interact with PtkA. Here, we report that DefA is crucial for *B. subtilis* ability to cope with oxidative stress. We demonstrate that the protective effect of DefA proceeds mainly via direct inhibition of the BY-kinase PtkA and does not involve the major known transcriptional responses to oxidative stress, dependent on the transcriptional regulators PerR, OhrR, and Spx. We also clarify the structural aspects of PtkA-DefA interaction, identifying the key interaction residues in DefA, and separate residues required for kinase inhibition. Point mutants of these residues impaired postoxidative stress recovery in vivo, corroborating the proposed mechanism.

## Results and Discussion

### DefA Residues 59–155 Are Essential for Interaction with PtkA.

**Fig. 1.**
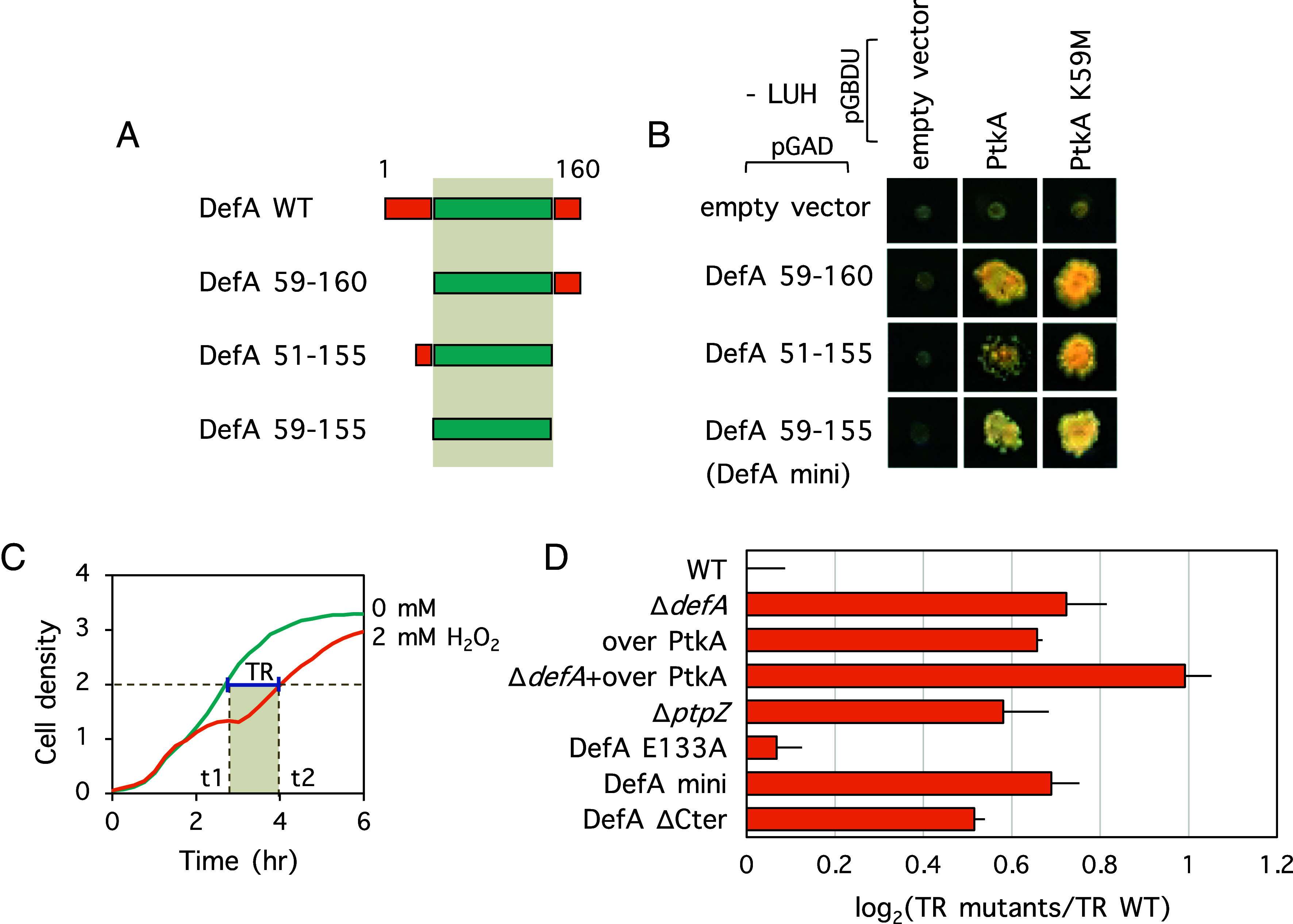
DefA interacts with PtkA and increases the resistance of *B. subtilis* to oxidative stress. (*A*) Schematic representation of DefA wild type and mutants tested in yeast two-hybrid. The minimal interaction domain is highlighted in gray. (*B*) Interaction between DefA variants and PtkA in yeast two-hybrid. *ptkA* and *ptkA* K59M were inserted into the plasmid pGBDU to obtain their translational fusion with the binding domain of the Gal4 regulator. Variants of *defA* were inserted into plasmid pGAD to obtain their translational fusion with the activating domain of the Gal4 regulator. Empty vectors were used as negative controls. Yeast *a* and *α* haploid strains expressing the binding domain and activating domain used proteins, respectively, were mixed to form diploids in which two proteins get coexpressed. The capability of protein–protein interaction was visualized by the ability of the diploid cells growing on –LUH selective medium. (*C*) The definition of time of recovery (TR). The capability of *B. subtilis* strains to recover from H_2_O_2_ treatment was indicated by TR, which is calculated as the difference in the time it takes to reach OD_600_ of 2 for the treated and nontreated cells. The shaded rectangle indicates the TR on the *x*-axis (t2-t1). (*D*) TRs of *B. subtilis* strains with H_2_O_2_ treatment. The change of TRs in mutants versus wild type is expressed as log_2_ ratio. The TR of the wild type is normalized as 1. Data are presented as mean + SD.

Bacterial translation begins with N-formylmethionine (fMet); hence, fMet becomes the N-terminal amino acid of most newly synthesized bacterial proteins. The N-formyl groups are removed posttranslationally by PDFs. DefA is recognized as a minor PDF in *B. subtilis*, with most peptide deformylation in the cell being carried out by its homologue, DefB (20). Our previous screening of the genomic-wide yeast two-hybrid library of *B. subtilis* with PtkA revealed a protein–protein interaction between DefA and PtkA (19). Native DefA is a protein with 160 amino acids. To identify the minimal DefA domain required for interaction with PtkA, we constructed several truncated versions of DefA, namely DefA 59–160, DefA 51–155, and DefA 59–155 (Fig. 1*A*). These were employed as “prey,” and PtkA was used as “bait” in a subsequent two-hybrid experiment. As shown in Fig. 1*B*, all three shorter versions of DefA interacted with PtkA, suggesting that the minimal region of DefA required for interaction with PtkA is situated in the region between the residues 59 and 155. This minimal interaction domain of DefA was named DefA-mini. Interestingly, a catalytically inactive version of PtkA, in which the key catalytic residue lysine 59 was substituted with methionine (PtkA K59M) (19), was fully capable of interacting with DefA (Fig. 1*B*), indicating that the interaction is independent from PtkA activity.

### DefA Protects *B. subtilis* from Oxidative Stress.

Next, we investigated the physiological relevance of the DefA-PtkA interaction. For this, we turned to a previous study by Nicolas et al. (21), which reported an exhaustive catalogue of transcriptional responses of *B. subtilis* over 104 different environmental and nutritional conditions. We examined this dataset, focusing on the respective expression patterns of genes encoding DefA, PtkA, and TkmA, a known activator PtkA (8). In most of the examined conditions, the expression of the three genes followed a similar pattern (*SI Appendix*, Fig. S1). However, under conditions of oxidative stress, sporulation, and germination, the expression patterns of *defA* diverged from *ptkA* and *tkmA*, which remained coupled. Given the known link between bacterial protein-tyrosine phosphorylation and oxidative stress (3, 4), we chose to examine the functional interaction of DefA and PtkA under these conditions. For our investigation, we constructed several *B. subtilis* strains using a replication-thermosensitive vector pMAD, to obtain seamless mutants. In the *B. subtilis* genome, *defA* and *fmt* form an operon and are transcribed from the same promoter. There are only four base pairs between the stop codon of *defA* and the start codon of *fmt,* which indicates an overlap of the ribosomal binding site of *fmt* and the 3′ end of the *defA* ORF (*SI Appendix*, Fig. S2*A*). To maintain the translation of *fmt* and minimize the polar effect, we kept the last 33 base pairs at the 3′ end of the *defA* ORF when constructing the *defA* deletion (*SI Appendix*, Fig. S2*B*). The strain *B. subtilis* Δ*defA* did not show any growth defect compared to the wild type in regular cultivation (*SI Appendix*, Fig. S2*C*). Next, we assessed the response of *B. subtills* to different concentrations of H_2_O_2_ in liquid cultures (*SI Appendix*, Fig. S3*A*). With 2 mM H_2_O_2_, we observed a very reproducible growth-slowing defect, which could be reliably quantified as the “time of recovery” (TR) (Fig. 1*C*). Using 2 mM H_2_O_2_ treatment, we recorded the growth response (*SI Appendix*, Fig. S3*B*) and quantified the TR (Fig. 1*D*) for all relevant mutant strains of *B. subtilis.* Deletion of *defA* resulted in a clear increase in TR, indicating increased sensitivity to H_2_O_2_. Point mutation *defA* E133A, which inactivates the catalytic site of DefA (22), had no significant effect on TR, indicating that PDF activity is not needed for the protective effect of DefA against oxidative stress. Mutations increasing tyrosine phosphorylation levels, such as overexpression of *ptkA* and inactivation of the phosphatase gene *ptpZ* increased in TR, in line with observations in *Mycobacterium tuberculosis* that tyrosine phosphorylation negatively affects cellular resistance to oxidative stress (23). The results indicated the potential involvement of DefA in PtkA signaling and oxidative stress responses.

### During Oxidative Stress, DefA Inhibits PtkA.

**Fig. 2.**
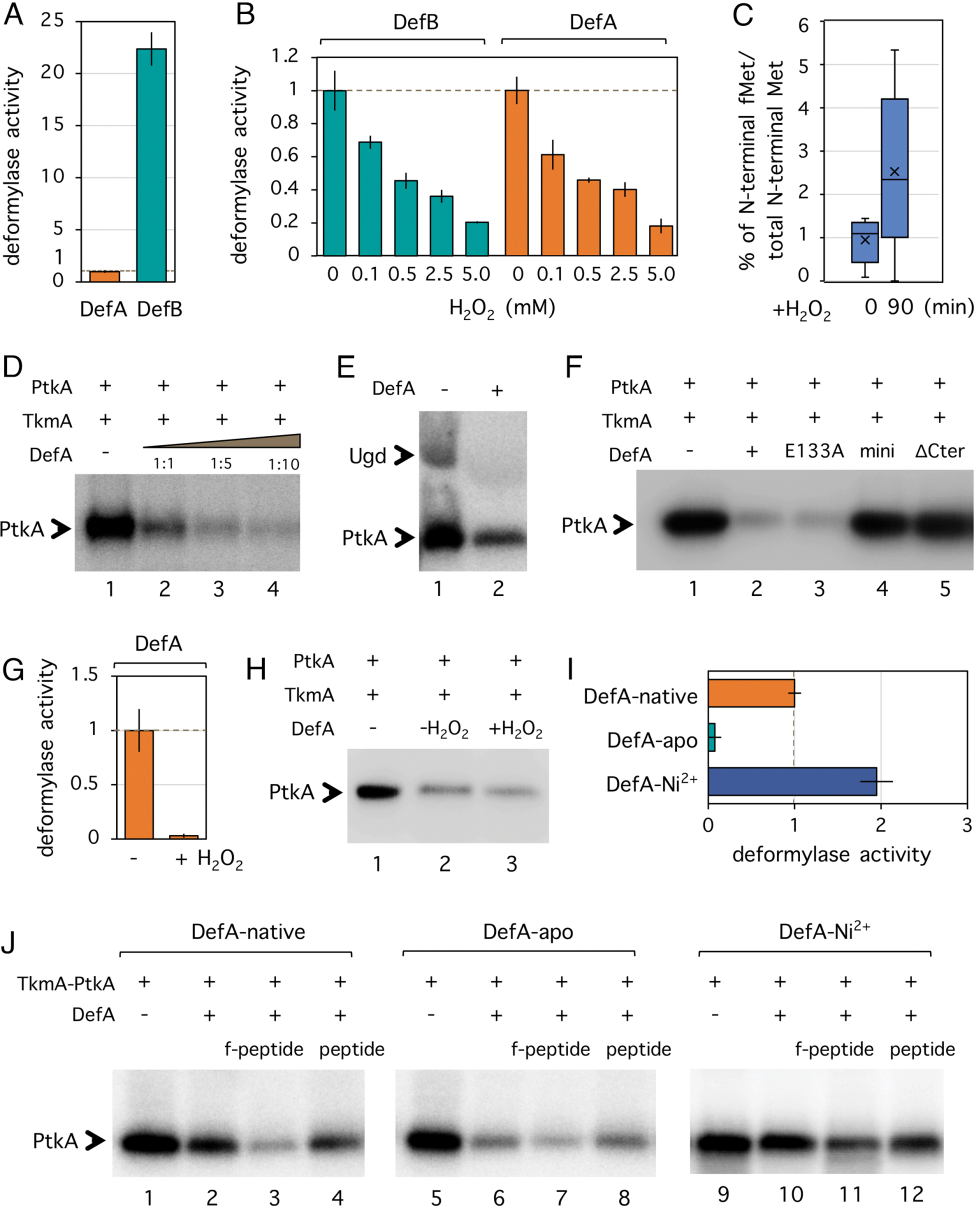
DefA inhibits PtkA phosphorylation activity during oxidative stress. (*A*) In vitro formylmethionine deformylase activity of DefA and DefB. Measurement was performed in reactions using N-Formyl-Met-Leu-Phe (fMLF) as the substrate. The activity of DefA was normalized as 1. Data are presented as mean + SD. (*B*) In vitro formylmethionine deformylase activity of DefA and DefB treated with different concentrations of H_2_O_2_: 0, 0.1, 0.5, 2.5, and 5 mM. Enzymatic activities were measured using fMLF as the substrate. Activities of DefB and DefA at an H_2_O_2_ concentration of 0 mM were normalized as 1, respectively. Data are presented as mean + SD. (*C*) In vivo accumulation of N-formylmethionine peptides in *B. subtilis* wild type after H_2_O_2_ treatment. The level of N-formylmethionine peptides is presented as a ratio of N-formylmethionine peptides versus total N-terminal peptides in the same sample. Eight fractions from each sample were measured. Data are presented by box plot with *P* < 0.05. (*D*) In vitro phosphorylation of PtkA in the presence of TkmA and DefA. Phosphorylation was performed in reactions containing ^32^P-γ-ATP. Protein concentration in reactions is described in *Materials and Methods*. The presence or absence of proteins is indicated as +/− above each lane. The ratio of DefA to TkmA in lanes 2, 3, and 4 is 1:1, 1:5, and 1:10, respectively. Phosphorylation signals were revealed by autoradiography. Bands corresponding to autophosphorylation of PtkA are indicated by an arrow. (*E*) In vitro phosphorylation of Ugd by PtkA in the presence of DefA. Ugd, PtkA, and TkmA were mixed in the absence (lane 1) or presence (lane 2) of DefA. Bands corresponding to phosphorylation of Ugd and PtkA are indicated by arrows. (*F*) In vitro phosphorylation of PtkA in the presence of DefA variants. The ratio of DefA to PtkA in lanes 2 and 5 was 1:10. (*G*) In vitro formylmethionine deformylase activity of DefA inactivated by H_2_O_2_ treatment. DefA (+H_2_O_2_): Crude extracted total protein was subjected to H_2_O_2_ treatment to inactivate DefA before purifying the protein by affinity chromatography. DefA (−H_2_O_2_): DefA purified from the same crude extract but without H_2_O_2_ treatment. (*H*) In vitro phosphorylation of PtkA in the presence of inactivated DefA. DefA (+H_2_O_2_) and DefA (−H_2_O_2_) are the same protein samples as shown in *G*. The ratio of DefA to PtkA in lanes 2 and 3 was 1:1. (*I*) In vitro formylmethionine deformylase activity of three forms of DefA: DefA-native (DefA-Fe^2+^), DefA-apo, and DefA-Ni^2+^. The activity of DefA-native was normalized as 1. Data are presented as mean + SD. (*J*) In vitro phosphorylation of PtkA in the presence of DefA, f-peptide (N-formylmethioninated peptides), and peptide (nonmodified peptides). The ratio of DefA to PtkA in lanes was 1:5.

PDFs such as DefA use single iron ions (Fe^2+^) as a prosthetic group, which binds the substrate directly (24). H_2_O_2_ and superoxide (O^2–^) are known to diminish deformylase activity by oxidation of the catalytic Fe^2+^ ion into the catalytically inactive Fe^3+^ or Fe^4+^ (25). To check this specifically for *B. subtilis* PDFs, we measured the in vitro PDF activity of DefA and DefB, with and without the H_2_O_2_ treatment. For this, 6×His tagged DefA and DefB were expressed in *E. coli* and purified. The in vitro enzymatic assay was performed using N-Formyl-Met-Leu-Phe (fMLF) peptides as substrates. The released free Met-Leu-Phe (MLF) was detected with fluorescamine, which reacts with primary amines and forms intensely fluorescent adducts. Consistent with the findings of Haas et al. (20), DefB was 20-fold more active than DefA (Fig. 2*A*). When treated with different concentrations of H_2_O_2_, both DefA and DefB showed a similar, concentration-dependent inactivation pattern (Fig. 2*B*). Although a direct comparison between this in vitro result and the in vivo experiment shown in Fig. 1*D* is not possible, it is probably safe to assume that the DefA and DefB PDF activity drops significantly in *B. subtilis* cells during oxidative stress. We tested this assumption in vivo, by estimating the total amount of cellular N-formylmethioninated peptides using mass spectrometry (Fig. 2*C*). Within 90 min of H_2_O_2_ treatment, the cellular levels of N-formylmethioninated peptides increased 2.5-fold, consistent with the assumption that PDFs get partially inactivated.

How can enzymatically inactive DefA help *B. subtilis* in coping with oxidative stress? We hypothesized that DefA interaction with PtkA could explain this effect. Since DefB did not interact with PtkA in any of the two-hybrid assays we performed, it was excluded from this line of investigation. To test our hypothesis, we investigated the impact of DefA-PtkA interaction on the kinase activity. We performed an in vitro phosphorylation assay based on incorporation of ^32^P into proteins from ^32^P-gamma-labeled ATP (8). Purified 6xHis-tagged DefA was incubated with PtkA and its activator TkmA, and all phosphorylated proteins were revealed by autoradiography (Fig. 2*D*). DefA was not phosphorylated by PtkA, but autophosphorylation of PtkA was inhibited in the presence of DefA, in dose-dependent manner. As far as we were able to ascertain, no similar protein inhibitors of BY-kinases have been reported. PtkA was previously shown to respond to different protein activators, MinD and SalA, both identified as PtkA interactants by yeast two-hybrid (19, 26). Since PtkA autophosphorylation and substrate phosphorylation are independent reactions, both dependent on ATP, we tested the effect of DefA on the principal substrate of PtkA, the UDP-glucose dehydrogenase Ugd. Ugd phosphorylation by PtkA was almost completely abolished in the presence of DefA (Fig. 2*E*). It is important to remember that the enzymatically inactive DefA E133A conferred full protection against oxidative stress in vivo (Fig. 1*D*). DefA E133A also exhibited full inhibition of PtkA autophosphorylation and Ugd phosphorylation by PtkA in vitro (Fig. 2*F* and *SI Appendix*, Fig. S4*A*). Hence, we concluded that PDF activity of DefA is not needed for inhibition of PtkA. In line with this, in vitro DefA inactivated by H_2_O_2_ (Fig. 2*G*) also retained its ability to inhibit PtkA-dependent phosphorylation (Fig. 2*H*). Since PDFs were previously reported to be unstable in the presence of oxygen (25, 27), it is probable that DefA became partially inactivated during our in vitro assays. To further clarify the impact of oxidation on DefA interaction with PtkA, we used several different forms of the enzyme. Oxygen-induced inactivation of PDFs is correlated with Fe^2+^ oxidation. By replacing Fe^2+^ with Ni^2+^ in the active site, PDFs can become fully insensitive to oxidation (28, 29). Conversely, a completely inactive form of PDF, known as apo-PDF, can be obtained by removing all ions from its active site. Hence, we purified and compared three forms of DefA: DefA-native which contains Fe^2+^ in its active site and is presumably partially oxidated, DefA-apo in which Fe^2+^ was extracted by EDTA, and DefA-Ni^2+^ which contains only Ni^2+^ in its active site. As shown in Fig. 2*I*, DefA-apo had almost no enzyme activity, while DefA-Ni^2+^ was fully active. The specific activity of the native DefA was in between the two extremes, indicating partial oxidation of iron in its active site. The three forms of DefA exhibited the capacity to inhibit PtkA which was inversely proportional to their enzyme activity, i.e., the inactive DefA-apo showed the strongest inhibitory effect (Fig. 2*J*). This indicated that inactive DefA is indeed the form that inhibits PtkA. Since intracellular N-formylmethioninated peptides accumulate under oxidative stress (Fig. 2*C*), we asked whether they could in any way contribute to the DefA mediated inhibition on PtkA phosphorylation. As shown in Fig. 2*J*, the presence of N-formylmethioninated peptides enhanced the DefA-dependent inhibition of PtkA, while nonmodified peptides had no effect. N-formylmethioninated peptides alone (in the absence of DefA) did not exhibit any inhibition on PtkA autophosphorylation (*SI Appendix*, Fig. S4*B*). Based on these finding, we propose that under conditions of oxidative stress, partially inactivated DefA, in conjunction with higher concentration of N-formylmethioninated peptides, inhibits PtkA kinase activity. This is consistent with in vivo results, since both the mutants that overexpression of *ptkA* (*SI Appendix*, Fig. S3*C*) and the deletion of *ptpZ* (encoding a phosphatase of PtkA), which increase tyrosine phosphorylation, have a detrimental effect on peroxide resistance (Fig. 1*D*). DefA inhibition of PtkA counters this effect. When *ptkA* is overexpressed in the absence of DefA, the H_2_O_2_ sensitivity gets further exacerbated (Fig. 1*D*).

### PtkA Is Likely to be the Principal Target of DefA-Dependent Resistance to H_2_O_2_ Stress.

It has been previously reported that BY-kinases can antagonize resistance to H_2_O_2_ stress (3, 4, 23). While our results suggest that DefA acts via inhibiting PtkA, it is possible that DefA could also act through other parallel pathways. When *B. subtilis* undergoes H_2_O_2_ stress, the mechanisms of sensing oxidants and inducing expression of protective proteins include several key transcriptional regulators: PerR, OhrR, and Spx. Their regulons encode the radical-scavenging enzymes, catalase, and alkyl hydroperoxide reductase, all of which directly help *B. subtilis* to cope with oxidative stress (30). To check this main oxidative stress response pathway, we examined the transcription of *katA, ohrA,* and *trxA* by quantitative reverse-transcription PCR (qPCR) in Δ*defA*, *ptkA* overexpression, and Δ*ptpZ* strains. The genes selected as markers of oxidative stress response are as follows: *katA* belongs to the PerR regulon, and its product is catalase 1, *ohrA* belongs to the OhrR regulon and its product is peroxiredoxin, and *trxA* belongs to the Spx regulon, and codes for thioredoxin. As shown in *SI Appendix*, Fig. S5*A*, the expression of *katA, ohrA,* and *trxA* in response to the H_2_O_2_ challenge in all examined strains (Δ*defA*, *ptkA* overexpression, and Δ*ptpZ*) was up-regulated to the same levels as in the wild type. We viewed the results in an alternative way to compare the expression level of selected marker genes in each strain without and with H_2_O_2_ treatment to that in the wild type, respectively. The expression level in the examined strains was comparable to that in the wild type (*SI Appendix*, Fig. S5 *B* and *C*). This suggested that the DefA protective effect does not involve PerR, OhrR, and Spx regulons. Phosphorylation of protein chaperone DnaK by PtkA had been previously shown to assist in stress survival (9). However, phosphorylation of DnaK was not affected by DefA (*SI Appendix*, Fig. S6), ruling out DnaK as a player in this mechanism.

**Fig. 3.**
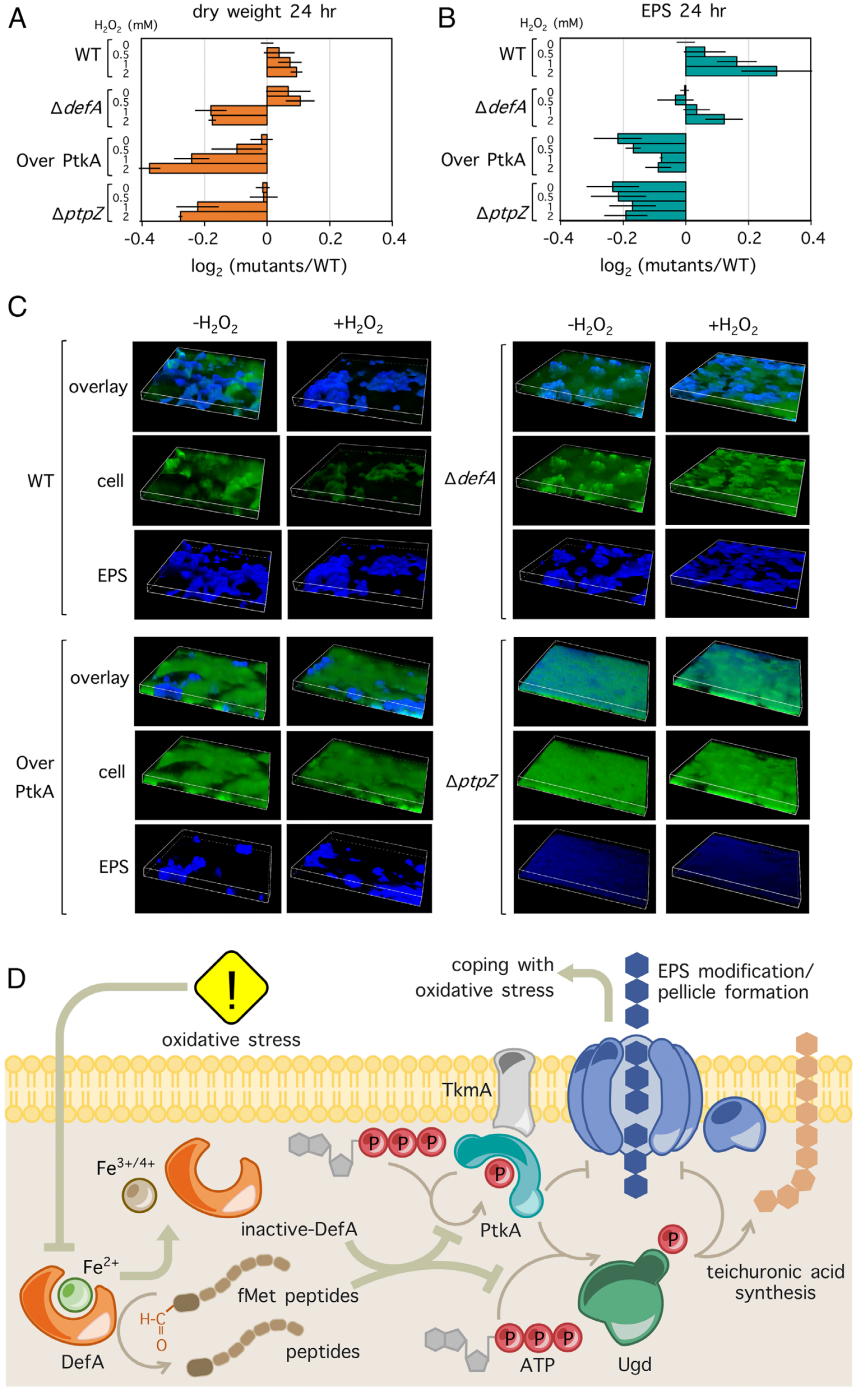
DefA is required for pellicle maturation and protection of bacteria during oxidative stress. (*A*) Measurement of dry weight per pellicle of wild-type, Δ*defA*, Over PtkA (overexpression *ptkA*), and Δ*ptpZ* strains. Pellicles were developed in the presence of different concentrations of H_2_O_2_ (0, 0.5, 1, and 2 mM) in Msgg medium for 24 h. The dry weight of the wild type at 0 mM H_2_O_2_ was normalized as 1. (*B*) Measurement of EPS per dry weight of wild-type, Δ*defA*, Over PtkA, and Δ*ptpZ* strains. (*C*) EPS distribution pattern in pellicles developed by wild-type, Δ*defA*, Over PtkA, and Δ*ptpZ* in the absence and presence of H_2_O_2_. Blue fluorescence indicates EPS stained with Alexa Fluor 594 Conjugated Concanavalin A (*Bottom* panel), green fluorescence indicates cells stained with SYTO 9 nucleic acid stain (*Middle* panel). Two channels are also shown in overlay (*Top* panel). The Z-axis range was 48.75 μm. (*D*) Schematic representation of the proposed hypothetical mechanism of DefA action on PtkA, helping to cope with oxidative stress in *B. subtilis*. Oxidative stress inactivates DefA by oxidation of ferrous ion, which dissociates from the enzyme. Inactivation of DefA (and DefB, not shown), leads to an increase of intracellular N-formylmethionine peptides. DefA, assisted by N-formylmethionine peptides, inhibits PtkA autophosphorylation and phosphorylation of Ugd. Excessive phosphorylation of PtkA and activity of Ugd (caused by phosphorylation) are both detrimental for EPS synthesis. Therefore, the inhibitory effect of DefA on PtkA facilitates EPS production and export, and pellicle/biofilm formation, which helps *B. subtilis* to cope with oxidative stress.

DefA inhibition of PtkA attenuated kinase autophosphorylation and phosphorylation of Ugd (Fig. 2 *D* and *E*). As mentioned before, phosphorylation/dephosphorylation of BY-kinases is important in biofilm formation/EPS synthesis. In addition, as a UDP-glucose dehydrogenase, Ugd converts UDP-glucose to UDP-glucuronate that is used as a precursor in teichuronic acid synthesis. PtkA phosphorylates Ugd and thus increases its enzyme activity (8). It has been indicated in a previous study that deletion of genes encoding UDP-glucose dehydrogenases did not affect complex colony formation in *B. subtilis* (15). Moreover, overexpression of these enzymes in *Bacillus licheniformis* antagonized EPS synthesis (31). Therefore, we asked whether this protective effect could involve the EPS synthesis. To test this, we examined the performance of Δ*defA, ptkA* overexpression, and Δ*ptpZ* in terms of biofilm formation during oxidative stress. Pellicle is a structure formed by *B. subtilis* cells at the air–liquid interface, which is used to monitor biofilm formation in liquid cultures. As shown in *SI Appendix*, Fig. S7*A*, pellicle developed by Δ*defA* exhibited the same architecture as the wild type in the regular MSgg medium, while pellicles formed by Δ*ptpZ* and *ptkA* overexpression strains were defective. This result confirmed the direct involvement of PtkA and PtpZ in biofilm formation, in agreement with previous findings (15). While DefA was not essential for pellicle development in normal conditions, Δ*defA* strain failed to develop the mature pellicle, showing a similar phenotype to the Δ*ptpZ* and *ptkA* overexpression strains, when increasing concentrations of H_2_O_2_ were added to the MSgg medium at the beginning of the experiment. To characterize this behavior further, we measured dry weight of pellicles and quantified the EPS produced by all strains (Fig. 3 *A* and *B*). Δ*defA,* ptkA overexpression, and Δ*ptpZ* strains exhibited less dry weight per pellicle compared with the wild type. EPS production was induced in the wild-type strain upon H_2_O_2_ treatment, in agreement with a previous study (18). Δ*defA* produced amounts of EPS similar to the wild type without H_2_O_2_ treatment, but it was not able to reach wild-type levels of EPS when challenged with H_2_O_2_. *ptkA* overexpression and Δ*ptpZ* strains produced less EPS both in the absence and presence of H_2_O_2_ compared with the wild type. Next, we visualized the EPS distribution pattern in pellicles grown for 24 h, using fluorescent microscopy (Fig. 3*C* and *SI Appendix*, Fig. S8*A*). EPS was stained by Alexa Fluor 594 Conjugated Concanavalin A, and cells were stained by 2 SYTO 9 nucleic acid stain. As shown in Fig. 3*C*, wild-type strain formed complex EPS structures in the absence of H_2_O_2_ and its EPS layer became thicker after H_2_O_2_ treatment, in which EPS forms a grid that wraps around cells (*SI Appendix*, Fig. S8*A*). While the EPS formed by Δ*defA* was similar to the wild type without oxidative stress, this strain was not able to form complex structures made by the wild type in the presence of H_2_O_2_. Mutant strains *ptkA* overexpression and Δ*ptpZ* also failed to form complex EPS structures in the absence and in the presence of H_2_O_2_. Of note, when pellicles were examined after a longer time period (48 h), cells were dying, and the complex structure was less pronounced, but the overall trend of differences in EPS production and EPS structure that were observed at 24 h still persisted (*SI Appendix*, Figs. S7 and S8*B*). Since our initial observations were made in planktonic cells (Fig. 1*D*), we also measured the EPS/dry weight in such conditions, to further verify the importance of EPS for protection against oxidative stress. All mutants exhibited wild-type levels of EPS in the absence of H_2_O_2_, at time point t1 (Fig. 1*D* and *SI Appendix*, Fig. S9*A*), while all of them produced sub-wild-type levels of EPS after H_2_O_2_ treatment at time point t2 (Fig. 1*D* and *SI Appendix*, Fig. S9*B*). We also examined the H_2_O_2_ resistance of preestablished biofilms. To do so, complex colonies, developed on Msgg plates for 24 h were soaked in H_2_O_2_ solutions for 2 h, and live and dead cells were visualized subsequently (*SI Appendix*, Fig. S10). Preestablished biofilms of the wild type and the Δ*defA* strain that form similar amounts of EPS in the absence of H_2_O_2_ (Fig. 3*B*) were equally resistant. By contrast, *ptkA* overexpression and Δ*ptpZ* strains, which contained less EPS, exhibited strongly impaired resistance. The observed defects in biofilm development and stress resistance of mutant strains were not caused by failure to trigger oxidative stress response, since the expression of *katA, ohrA,* and *trxA* in all strains (Δ*defA*, *ptkA* overexpression, and Δ*ptpZ*) was comparable to that in the wild type, at all time periods examined (*SI Appendix*, Fig. S11). This indicated that the regulation of the general oxidative stress response is not affected by DefA and DefA-mediated PtkA inhibition. These results were consistent with PtkA being the principal target of DefA protective action during oxidative stress. Therefore, we propose that inhibition of PtkA autophosphorylation and phosphorylation of Ugd helps cells to cope with oxidative stress by producing a protective EPS layer. A hypothetical mechanism based on our current data is outlined in Fig. 3*D*. While our experiments excluded the involvement of PerR, OhrR, and Spx regulons and other PtkA substrates such as DnaK in this mechanism, presently unknown other modes of action of DefA cannot be excluded.

### Interaction with PtkA and Kinase Inhibition Require Separate Key Residues of DefA.

**Fig. 4.**
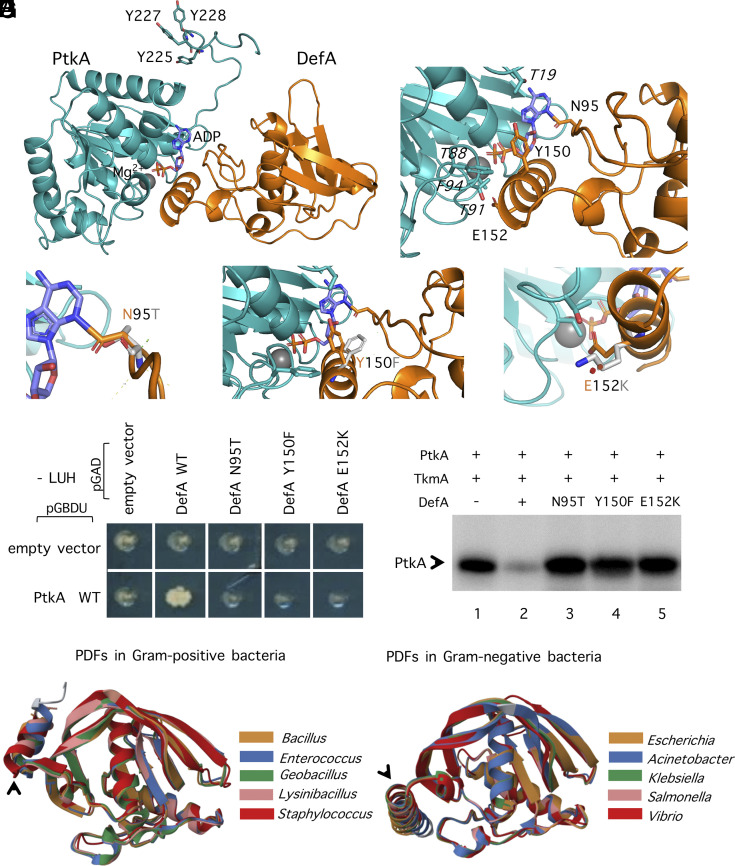
Structural features of DefA that are necessary for interaction with and inhibition of PtkA. (*A*) Structural analysis of the DefA-PtkA complex. Computational docking of DefA interacting with PtkA is shown, with DefA in orange and PtkA in teal. The ADP is shown in slate blue sticks, while the magnesium ion is represented with a gray sphere. (*B*) DefA residues engaging in direct interaction with the active site of PtkA and its (auto)phosphorylation activity. The interacting residues from both DefA and PtkA are shown in stick representation. (*C*) Point mutation N95T, in stick representation, showing how a smaller threonine side chain would abolish the interaction of DefA with PtkA. (*D*) Point mutation Y150F, shown as sticks, showing how the mutation would abolish the polar contact that DefA established through the OH group of Y150. (*E*) E152K mutation shown in sticks, where an oppositely charged and much larger lysine would clash with the PtkA loop, breaking the interaction between DefA and PtkA. (*F*) Interaction between DefA point mutants and PtkA in yeast two-hybrid. PtkA wild type was expressed from the plasmid pGAD, as a translational fusion with the activating domain of the Gal4 regulator. DefA wild type, DefA N95T, Y150F, and E152K were expressed from the plasmid pGBDU, as translational fusions with the binding domain of the Gal4 regulator. Empty vectors were taken as negative controls. Diploids were formed from *a* and *α* haploid strains expressing the binding domain- and activating domain-fused proteins respectively. (*G*) In vitro phosphorylation of PtkA in the presence of DefA variants. Phosphorylation was performed with ^32^P-γ-ATP and protein concentration in each reaction is described in *Materials and Methods*. The presence or absence of proteins is indicated as +/− above each lane. The ratio of DefA variants to PtkA in lanes 2–5 was 10:1. Phosphorylation was revealed by autoradiography. Bands corresponding to autophosphorylation of PtkA are indicated by arrow. (*H*) Structure alignment of four PDFs from gram-positive bacteria with *B. subtilis* DefA and (*I*) four PDFs from gram-negative bacteria with *E. coli* Def. Color code is provided in the figure. Uniprot access numbers for the used proteins: *B. subtilis* P94462, *Enterococcus casseliflavus* A0A1V8ZHL4, *Geobacillus thermoleovorans* A0A098L406, *Lysinibacillus antri* A0A432LGN4, *Staphylococcus petrasii* A0A380FZW5, *E. coli* P0A6K3, *Acinetobacter baumannii* B0VNL8, *K. pneumoniae* A0A2X3E363, *Salmonella typhimurium* Q8ZLM7, and *Vibrio vulnificus* Q8DDE3. Images of protein structures were created by Mol* (35, 36).

To identify key residues of DefA that are important for DefA-PtkA interaction, in silico docking was performed (Fig. 4*A*). Protein structures of PtkA and DefA were predicted by Alphafold 2 (32). The docking revealed three amino acids of DefA with most crucial contribution to the interaction with PtkA: aspartic acid 95, tyrosine 150, and glutamic acid 152 (Fig. 4 *B*–*E*), where Asn95 interacted with the head group of bound ADP, Tyr150 interacted with Thr19, Phe94, and ADP, while Glu152 interacted with Thr88 and Thr91, on the loop covering the active site of PtkA (Fig. 4*B*). We constructed point mutated DefA in which Asn95, Tyr150, and Glu152 were substituted by threonine, phenylalanine, and lysine, respectively. In prediction, N95T mutation would abolish the binding to the ADP when a longer Asn is replaced by a shorter Thr (Fig. 4*C*); the Tyr 150 when replaced by Phe turns its side chain toward DefA, abolishing the polar interaction formed by the Tyr to the PtkA (Fig. 4*D*); the Glu at position 152 is critical in forming h-bonds with PtkA, where a mutation to an opposite charged Lys, would break the interaction which also, is longer and would clash with the PtkA loop heading toward DefA (Fig. 4*E*). A yeast two-hybrid assay demonstrated that all three point mutations abolished the ability of DefA to interact with PtkA in vivo (Fig. 4*F*). Consequently, DefA N95T, Y150F, and E152K also lost their capacity to inhibit PtkA in the in vitro phosphorylation assay (Fig. 4*G*). Interestingly, DefA mini, which contains all key interaction residues, and interacts with PtkA (Fig. 1*B*), was impaired in terms of H_2_O_2_ protective effect in vivo (Fig. 1*D*). It was also unable to inhibit PtkA phosphorylation reaction in vitro (Fig. 2*F* and *SI Appendix*, Fig. S4*A*). This implies that inhibition requires separate structural features in addition to the ones required for DefA-PtkA interaction. As shown in Fig. 4*A*, a C-terminal α-helix of DefA interacts extensively with the ATP-binding pocket of PtkA. This helix is highly negatively charged. Charge-based interactions are usually of high affinity and are known to form tight bonds, especially in case of inhibition of the binding partner (33, 34). In explaining the effect of the DefA C-terminal α-helix on PtkA, we based our reasoning on the resolved structure of CapB, a BY-kinase from *Staphylococcus aureus* (6). CapB is activated by a cognate activator protein CapA. The C terminus of CapA possesses an α-helix, followed by a β-sheet, which both interact with the ATP binding site of CapB. This C-terminal tail of CapA stabilizes the ATP binding site and increases the affinity of CapB for ATP, resulting in increased kinase activity. The C-terminal tail of DefA contains a similar α-helix but lacks the β-sheet. Therefore, it presumably interacts with the ATP binding site of PtkA nonproductively. The tight interaction of the DefA C-terminal α-helix with the ATP binding site of PtkA blocks access to TkmA, which normally activates PtkA. This hypothesis is in line with the observed dose-dependent inhibition of PtkA by DefA, titrating out the available TkmA (Fig. 2*D*). To test this assumption, we constructed a DefA mutant, DefA ΔCter, in which the C-terminal α-helix of DefA was deleted. To maintain the capability of DefA to interact with PtkA, only amino acids 156–160 were removed. DefA ΔCter did not inhibit PtkA in vitro (Fig. 2*F* and *SI Appendix*, Fig. S4*A*). In vivo, the *B. subtilis* strain with *defA* replaced by *defA* ΔCter showed the same TR as Δ*defA* (Fig. 1*D*), confirming that the C-terminal α-helix of DefA is crucial for inhibition of PtkA.

### Inhibition of BY-Kinases by PDFs Is Probably a Common Feature in Gram-Positive Bacteria.

DefA-type PDFs are widely distributed in both gram-positive and gram-negative bacteria (37), and even mitochondria in eukaryotic organisms. As mentioned previously, BY-kinases and their activators have different protein architectures in gram-negative and gram-positive bacteria: they were found in the forms of a single polypeptide in gram-negative bacteria, while two separate proteins in gram-positive bacteria. This could be expected to affect the potential of BY-kinases to interact with PDFs. The overall three-dimensional structures of *E. coli* Def and *B. subtilis* DefA align well, except for the C-terminal α-helix (*SI Appendix*, Fig. S12*A*). *B. subtilis* C-terminal α-helix is shorter, more exposed to the solvent, and its axis is rotated by 90° with respect to its *E. coli* counterpart (*SI Appendix*, Fig. S12*B*). This difference can be further illustrated by aligning several PDFs from gram-positive and gram-negative bacteria, all of which exhibited very similar folds (Fig. 4 *H* and *I*). In gram-positive bacteria, the BY-kinase activators are present as a separate polypeptide, which could create more opportunities for BY-kinases to dissociate from cognate activators, and interact with other regulatory proteins, such as DefA. Given the similarity of structure of DefA-like PDFs in gram-positive bacteria, the DefA-dependent inhibition of BY-kinases could possibly occur in other bacteria.

## Conclusion

Eukaryotic and prokaryotic organisms employ different types of cell signaling in response to oxidative stress. Under such conditions, protein-tyrosine phosphorylation gets boosted in eukaryotic organisms, while it diminishes in prokaryotes. In this study, we report a regulatory mechanism used by *B. subtilis* to reduce tyrosine phosphorylation and help survival upon oxidative stress. The BY-kinase PtkA is inhibited by oxidation-inactivated DefA, a minor PDF, which results in a decrease of PtkA autophosphorylation and reduced phosphorylation of one of its substates, Ugd. Since the activity of Ugd can inhibit synthesis of EPS, its inactivation improves cell survival under oxidative stress. The structural motifs required for PDF-BY-kinase interaction are present in other gram-positive bacteria, indicating that this mechanism could be of general relevance.

## Materials and Methods

Please refer to *SI Appendix*, *Supplemental Materials and Methods* for details.

### Growth Condition and Strain Construction.

*B. subtilis* strain NCIB3610 (*comI*^-^) from BGSC (*Bacillus* Genetic Stock Center) with BGSCID 3A38 was used in this study and subjected to the following construction of genetic mutations. PCR primers used for plasmid and strain construction are listed in *SI Appendix*, Table S1. Details of plasmid and strain construction are described in *SI Appendix*.

### Yeast Two-Hybrid.

Yeast two-hybrid was performed as described in *SI Appendix*.

### Growth Measurement of *B. subtilis* Strains Upon Oxidative Stress.

Growth phenotype of *B. subtilis* wild-type and relevant strains with H_2_O_2_ treatment was measured as described in *SI Appendix*.

### Production and Purification of Heterologous Proteins.

The 6×His-tagged DefA, DefB, PtkA, TkmA, Ugd and Dnak was purified as described in *SI Appendix*.

### In Vitro Protein Phosphorylation Assay.

Protein phosphorylation was performed in vitro with [γ-^32^P]-ATP and visualized by autoradiography. Details are provided in *SI Appendix*.

### Deformylase Activity Assay.

In vitro deformylase activity was measured as described in *SI Appendix*.

### Quantification of the fMet Level in *B. subtilis* by Proteomics.

The level of N-formylmethioninated and N-methioninated peptides was quantified by mass spectrometric analysis as described in *SI Appendix*.

### Pellicle Formation.

The *B. subtilis* wild-type and relevant strains were subjected to pellicle formation as described in *SI Appendix*.

### Pellicle Weight Assay.

The dry weight of pellicles was measured as described in *SI Appendix*.

### EPS Analysis of Biofilms.

The extraction and measurement of EPS were performed as described in *SI Appendix*.

### Microscopic Analysis of Biofilms.

The biofilms developed by *B. subtilis* wild-type and relevant strains were stained and then visualized by a laser scanning confocal microscope as described in *SI Appendix*.

### Biofilm H_2_O_2_ Resistance Assay.

H_2_O_2_ resistance of preestablished biofilms was performed on complex colony as described in *SI Appendix*.

### qPCR.

qPCR was performed as described in *SI Appendix*.

### Computational Protein Structure Prediction.

Protein structure was predicted as described in *SI Appendix*.

## Supplementary Material

Appendix 01 (PDF)

## Data Availability

All study data are included in the article and/or *SI Appendix*.
