# Supplementary material for: Connection between protein-tyrosine kinase inhibition and coping with oxidative stress in Bacillus subtilis
Source: Proc Natl Acad Sci U S A. 2024 Jun 10;121(25):e2321890121. doi: 10.1073/pnas.2321890121 (PMC11194573; doi:10.1073/pnas.2321890121)
Supplement: Supplementary file 1 — Appendix 01 (PDF) [file pnas.2321890121.sapp.pdf]

Supporting Information for

Connection between protein-tyrosine kinase inhibition and coping with oxidative stress in *Bacillis subtilis*

Lei Shi, Abderahmane Derouiche, Santosh Pandit, Meshari Alazmi, Magali Ventroux, Julie Bonne Køhler, Marie-Francoise Noirot-Gros, Xin Gao, Ivan Mijakovic

Corresponding author:

Ivan Mijakovic, e-mail: ivan.mijakovic@chalmers.se

This PDF file includes:
Supplemental Materials and Methods
Figures S1 to S12
Table S1
Supplemental references

Supplemental Materials and Methods

Growth condition and strain construction

Cells were grown routinely in LB medium at 37°C, with addition of ampicillin (100 µg/ml), kanamycin (25µg/ml) for *E. coli*, erythromycin (1 µg/ml), chloramphenicol (5 µg/ml) for *B. subtilis*, when required. *E. coli* strain NM522 was used for pQE30-derived plasmid construction, DH5α was used for pMAD and pBS1C- derived plasmid construction. *E. coli* strain M15 (GroEL/S) was used for heterologous protein production and purification. *B. subtilis* strain NCIB3610 (*comI*$^{-}$) from BGSC (*Bacillus* Genetic Stock Center) with BGSCID 3A38 was used in this study and subjected to the following construction of genetic mutations.

Oligos listed in Supplementary Table S1 were used to amplify respective DNA fragments from *B. subtilis* NCIB3610 genomic DNA by PCR. For heterologous protein production, all PCR fragments were cloned into pQE30 (Qiagen) to obtain 6×His-tag fusion proteins. For *defA* wild type, mini, ΔCter and *defB*, they were amplified by their oligo pairs pQE_gene_fd and pQE_gene_rs, respectively. For DefA Y150F, oligo pair pQE_defA_fd and pQE_defA150_rs were used to amplify the DNA fragment. To obtain *defA* N95T and E133A, oligo pairs pQE_defA_fd and pQE_defA95/133_rs, pQE_defA95/133_fd and pQE_defA_rs, were used to amplify two partially overlapping DNA fragments containing parts of *defA* with point mutated sites, then they were joint by joint-PCR through pQE_defA_fd and pQE_defA_rs, to obtain full length *defA* with point mutated sites. The construction of *B. subtilis* strain NCIB3610 (*comI*$^{-}$) markerless mutations at the natural locus was performed by vector pMAD. For deletion of *ptpZ*, two DNA fragments containing upstream and downstream fragments of *ptpZ* ORF were obtained by oligo pairs pMAD_ptpZ_up_fd and pMAD_ptpZ_up_rs, pMAD_ptpZ_dw_fd and pMAD_ptpZ_dw_rs, then they were joint by joint PCR through pMAD_ptpZ_up_fd and pMAD_ptpZ_dw_rs. To delete *defA*, two DNA fragments containing flaking region of *defA* ORF were obtained by oligo pairs pMAD_defA_fd and pMAD_defA_up_rs, pMAD_defA_dw_fd and pMAD_defA_rs. They were joint by joint PCR using pMAD_defA_fd_eco and pMAD_defA_rs_bam. For *defA* E133K, two DNA fragments containing flaking region of *defA* and also partially overlapping parts of *defA* with point mutated sites were amplified by oligo pairs pMAD_defA133_fd and pMAD_defA133_up_rs, pMAD_def133_dw_fd and pMAD_def133_rs. By pMAD_def133_fd_eco and pMAD_133_rs_bam, two fragments were PCR-jointed. For DefA ΔCter, a similar strategy as the construction of *defA* E133A was employed. Two DNA fragments were amplified by pMAD_pm_defA_fd and pMAD_pm_def-detlaC_up_rs, pMAD_pm_def-deltaC_dw_fd and pMAD_pm_defA_rs, then they were PCR-jointed by pMAD_pm_defA_fd_eco and pMAD_pm_defA_rs_bam. To construct *defA* mini, three partially overlapping DNA fragments containing upstream flanking region of *defA*, *defA* mini and downstream flanking region of *defA* were amplified by oligo pairs pMAD_defA-mini_fd and pMAD_defA-mini_up_rs, pMAD_defA-mini_mid_fd and pMAD_defA-mini_mid_rs, pMAD_defA-mini_dw_fd and pMAD_defA-mini_rs, then they were joined by joint-PCR through pMAD_defA-mini_fd_eco and pMAD_defA-mini_rs_bam. All obtained PCR products were inserted into vector pMAD. To construct PtkA overexpression strain, promoter physpank was amplified by physpank_fd_new and physpank_rs, then inserted into pBS1C by EcoRI and SpeI. A BglII site was included in physpank_rs to be introduced into the resulting plasmid pBS1C-physpank. ORF of *ptkA* was amplified by oligo pairs physpank_ptkA_fd and physpank_ptkA_rs, then inserted into pBS1C-physpank by BglII and XhoI. For Yeast-two-hybrid, *defA* variants were amplified from pQE30-derived plasmid as templates by pGAD-C1_defA_fd and pGAD-C1_defA_rs, then inserted into pGAD-1C by EcoRI and BamHI.

Yeast-two-hybrid

The screening of the genomic yeast two-hybrid library of *B. subtilis* with PtkA K59M was performed as described previously (1). The validation of interaction between DefA minimal domain of interaction

with PtkA was performed as followed: the DNA fragments encoding the different domains of DefA previously identified in Y2HB screens with the bait protein PtkA, were PCR amplified and re-introduced into the pGAD vector as described elsewere (1). The PJ69-4*a* and *α* yeast strains carrying the baits PtkA and the preys DefA, respectively, were mated and diploids were first selected onto selective media (-LU). The interaction phenotypes were assayed for their ability to grow onto –LUH and –LUA (2). Interactions were considered as specific as they were reproduced at least twice and were not associated with autoactivation nor stickyness of the prey proteins as described (1).

Growth measurement of *B. subtilis* strains upon oxidative stress

Growth of *B. subtilis* strains was measured in BioLector ® I high-throughput microbioreactor (M2P Labs) with flower 48-well plates (M2P Labs). Cells from single colonies were cultivated in LB medium at 37°C with shaking at 200 rpm in incubator until OD_{600} reached 0.5, then diluted into fresh medium to OD_{600} 0.05, then loaded in 48 well FlowerPlate® (M2P Labs). Plates were incubated in BioLector ® I at 37 ℃ at 1000 rpm. H_2O_2 was added into cell culture when OD_{600} reaches 0.6. Measurement of growth was taken every 20 min.

Production and purification of heterologous proteins

Heterologous protein production and purification were performed as described previously (3). In brief, protein induction was started by addition of 1 mM IPTG to cell culture when OD_{600} reached 0.6. After 3 hours of induction, cells were collected and disrupted by sonication. The 6×His-tagged proteins were purified through Ni-NTA (Qiagen) and then desalted on PD-10 columns (Cytiva) before subjected to later assays.

To obtain DefA that is inactivated by H_2O_2, protein was induced by IPTG, then cells were spin down and disrupted by sonication as described previously. After sonication, cell lysate was subjected to centrifugation at 12000 rpm for 10 min. The obtained supernatant was split into two parts: one was incubated with 50 mM H_2O_2 for 30 min in ice to inactive DefA, the other was incubated with 0.5 mM $FeCl_2$ for 30 min in ice as control. After incubation, protein was purified through Ni-NTA (Qiagen) as described previously.

DefA-apo was obtained as described previously (4). DefA eluted from Ni-NTA column was incubated with 2 mM EDTA at final concentration in buffer containing 50 mM Tris pH 7.5, 100 mM NaCl, 5 mM $MgCl_2$, 5% glycerol for 30 min at room temperature to remove Fe^{2+}, then subjected to PD-10 columns to be desalted. DefA-Ni^{2+} was obtained as described previously (5). EDTA incubated DefA was mixed with equal volume of 3 mM $NiSO_4$ at 4°C for 1h, then subjected to PD-10 columns.

***In vitro* protein phosphorylation assay**

In vitro protein phosphorylation assay was performed as described previously (3). Proteins subjected to protein phosphorylation events were incubated at 37°C for 1 hour in a buffer containing 50 mM Tris pH 7.5, 100 mM NaCl, 5 mM $MgCl_2$, 5% glycerol. Protein concentration was as following: PtkA 2.5 µM, TkmA 2.5 µM, 7.5 µM Ugd and DefA 2.5 to 25 µM when ratio of PtkA to DefA was from 1:1 to 1:10, DefA variants 25 µM. 4 mM of peptide (Met-Ala-Ser) or f-peptide (N-Formyl-Met-Ala-Ser) was included into reaction when required. To analyze DnaK phosphorylation, 1 µM PtkA, 1 µM TkmA and 3 µM DnaK were mixed in the phosphorylation reaction. Reactions were started by addition of 50 mM ATP containing 20 mCi $mmol^{-1}$ [γ-^{32}P]-ATP and stopped by addition of SDS-protein loading dye and boiling at 100°C. Proteins were separated by 12% SDS-polyacrylamide gel. The radioactive phosphorylated proteins were visualized by auto-radiography using a phosphorimager Typhoon FLA7000 (Cytiva Amersham).

Deformylase activity assay

In vitro deformylase activity was measured as described previously (6). Reactions with a final volume of 100 µL containing 50 mM Tris, pH 7.5, 1 µM of DefA or 0.1 µM DefB, 0.7 M KCl, 0.035% Brij, and 4 mM N-Formyl-Met-Leu-Phe (Merck) were incubated at 37°C for 30 min, then stopped by the addition of 50 µL of ice cold 0.2 M sodium borate, pH 9.5. The free amino group of the product (Met-Leu-Phe) was detected by addition of 50 µL fluorescamine (0.2 mg/ml in dry dioxane) to each reaction. Fluorescence was measured in FLUOstar Omega plate reader (BMG LABTECH) with the excitation wavelength at 390 nm and the emission wavelength at 465 nm.

Quantify level of fMet in *B. subtilis* by proteomics

Protein extraction and MS sample preparation

Cell pellets were resuspended in lysis buffer (7 M urea, 100 mM Tris-HCl (pH 8.5), 1 % Triton-X100, 5 mM Tris (2-carboxyethyl) phosphine hydrochloride, 30 mM chloroacetamide, 10 U/mL DNase, 1 mM $MgCl_2$, 0.5 % benzonase (Merck Millipore), 1× Complete mini EDTA-free protease inhibitor cocktail (Roche)) and sonicated 8 × 30 sec using a cup horn sonicator at 50% amplitude. Lysates were clarified by centrifugation at 27000 g for 1 hour and the concentration of recovered protein was determined by a Bradford assay.

For protein digestion, 100 µg per sample was concentrated onto 30-kDa cut-off filter units (Millipore). Filter units were washed four times with 200 µl of UA buffer (8 M urea, 100 mM Tris-HCl pH 8.0), and proteins were alkylated with 30 mM chloroacetamide resuspended in UA for 20 min in the dark. Samples were equilibrated twice with 200 µl of 50 mM Tris pH 8.0, and digested with Trypsin/LysC (Promega, 1:50 enzyme-to-protein ratio) for 16 hours at 37°C. Resulting peptides were then fractionated into eight fractions using a High pH Reversed-Phase Peptide Fractionation Kit (Pierce).

Mass spectrometric analysis

For proteomic analysis, fractionated and desalted peptides were injected into an Orbitrap Exploris 480 mass spectrometer (Thermo Scientific) coupled to a CapLC system (Thermo scientific). Peptides were initially captured at a flow of 10 µL/min on a precolumn (µ-precolumn C18 PepMap 100, 5µm, 100Å) and then seperated on a 15 cm C18 easy spray column (PepMap RSLC C18 2 µm, 100Å, 150 µm ×15 cm) at a flow rate of 1.2 µL/min. The gradient was 4% to 76% acetonitrile in water over a period of 60 min. The mass spectrometer was operated in data dependent acquisition mode with the following settings: MS-level scans were performed with Orbitrap resolution set to 120,000 at a scan range (m/z) of 375-1500; normalized *AGC target of 300*%; maximum injection time set to Auto; intensity threshold at 5.0×10^3 and dynamic exclusion of 25 sec. Data-dependent MS2 selection was performed in Top 20 Speed mode with HCD collision energy set to 40%; isolation window at 1.3 m/z; normalized AGC target at 75% and maximum injection time set to 30 ms.

The raw files were analyzed with the Proteome Discoverer software (2.4) and spectra were searched against a *B. subtilis* 168 proteome database (UniProtKB, ID UP000001570, downloaded October 2021) with the following settings: Carbamidomethylation of Cys residues was set as a fixed modification, while variable modifications searched for were, formylation or acetylation of protein N-termini, and oxidation of Met residues. Trypsin/LysC was set as the digestion enzyme and two missed cleavages were allowed. Precursor mass tolerance was set at10 ppm; fragment mass tolerance at 0.02 Da; minimum peptide length at 7; maximum peptide length at 144; and the false-discovery rate (FDR) was set at 0.01 %.

Pellicle formation

For pellicle formation, cells from single colonies were cultured in LB medium at 37°C with shaking at 200 rpm in incubator until OD_{600} reached 0.5, then diluted 200-fold into 2 ml MSgg media in a 24-well

plate. After incubation 24 h and 48 h at 37°C, images of the pellicles were captured using Bio-rad ChemiDoc™ Imaging Systems.

Pellicle weight assay

Pellicle weight assay was performed as described previously (7). In brief, pellicles were grown in 6-well plates for 24 h and 48 h at 37°C. Then biofilm including medium were taken out, spin down, dried, and weighted.

EPS analysis of biofilms

Pellicles were grown in Msgg medium for 24 h and 48 h and collected for biochemical analysis. Briefly, biofilms formed in 24 well plates were collected in 5 mL of 0.89% of NaCl and washed three times (5000 g for 20 min) with sterile water. The washed biofilms were lyophilized and weighed to determine the biomass. The component of extracellular polymeric matrix was extracted using 1 N sodium hydroxide (100 µL/mg biomass for three times). The pH of extract was neutralized by using 1N HCl. The component of extracellular matrix was precipitated by overnight incubation of neutralized extract with 95% of ice-cold ethanol. The precipitate was later washed 3 times (5000 g for 20 min) with 75% of ethanol and dried at room temperature. The dried precipitate was dissolved with 1N sodium hydroxide for the quantification of polysaccharides, protein, and DNA in the biofilm matrix. The polysaccharide content in the biofilm was quantified by using a phenol-sulfuric acid assay as described previously (8).

Microscopic analysis of biofilms

For confocal laser scanning microscopy examination, extracellular polysaccharides in 24 h and 48 h biofilms was labelled with 50 µg/mL of Alexa Fluor 594 Conjugated Concanavalin A (590/617 nm; Molecular Probes) for 2 h (9). The bacterial cells in biofilms were stained with 2.5 µm of SYTO 9 nucleic acid stain (485/535 nm; Molecular Probes) for 30 min (10). The stained biofilms were transferred to a glass slide and laser scanning confocal microscope imaging of the biofilms was performed using Nikon Ti-E A1+.

Biofilm H_2O_2 resistance assay

H_2O_2 resistance assay of pre-established biofilm was performed on complex colony as described previously (11, 12). For complex colony formation, *B. subtilis* cells from single colonies were grown in LB medium at 37°C with shaking at 200 rpm until OD600 reached 0.5, then 10 µL of the culture was spotted onto Msgg plate with 1.5% agar and grown for 24 h at 37 ℃. The complex colony including the underlying agar was soaked into H_2O_2 solution with different concentration (0, 100, 200 and 500 mM) for 1 h. Then cells were scraped from underlying agar, collected, washed and resuspended in phosphate-buffered saline (PBS). The LIVE/DEAD BacLight Bacterial Viability Kit (ThermoFisher) was used to visualize the survival of cells. Images were captured by a LeicaCTR4000 inverted microscope.

Quantitative reverse-transcriptional PCR (qPCR)

For qPCR of *B. subtilis* strains in planktonic form, cells were cultured as described for Growth measurement in BioLector ® I high-throughput microbioreactor (M2P Labs) with flower 48-well plates (M2P Labs). Samples were taken at 0 min and 90 min after H_2O_2 treatment. For qPCR of *B. subtilis* strains in biofilm form, cells were cultured in Msgg medium with 0 and 2 mM H_2O_2 for 6, 24 and 48 h. Total RNA extraction was performed through RNeasy Mini Kit (Qiagen), and cDNA was reverse-transcribed through QuantiTect Reverse Transcription Kit (Qiagen). qPCR was performed with DyNAmo ColorFlash SYBR Green qPCR Kit (Thermo Scientific) in Agilent Technologies Mx3005P system. Primers are listed in Appendix Table S1.

Computational protein structure prediction

Computational protein structure prediction was performed using Alphafold2 (13). Molecular docking studies were performed using the ClusPro automated docking server (14). PDB ID 2VED (15) was used to model the ADP and Mg in the PtkA protein model. Further energy minimization for apo-PtkA, PtkA with ADP and Mg, DefA, DefA-mini, PtkA:DefA complex, PtkA:DefA-mini complex was performed using Yasara energy minimization module (16). Structural analyses were performed using PyMOL (www.pymol.org).

Def protein structures were predicted by Alphafold2 (13). Alignment of multiple protein structures was performed by Pairwise Structure Alignment, jFATCAT (flexible) (17). Images of protein structures were created using Mol* (RCSB.org) (17, 18).

Supplemental Figures and their legends

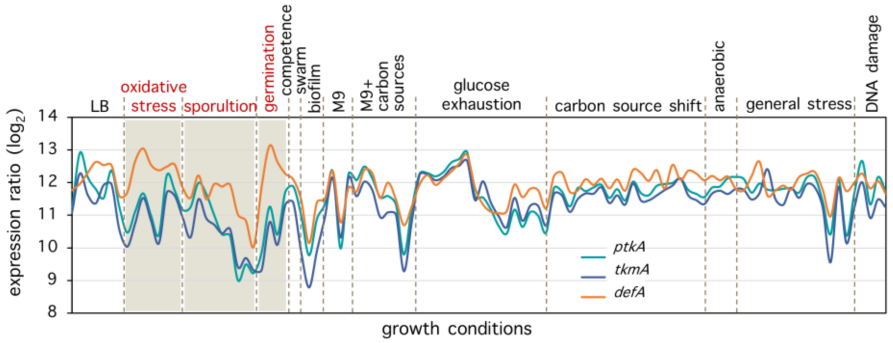

Fig. S1. Condition-dependent transcriptome of *B. subtilis*. Transcription profiles of *defA*, *ptkA* and *tkmA* in 104 reported conditions are shown in orange, blue and purple, respectively. Conditions where *defA* expression pattern deviates from that of *ptkA* and *tkmA* are highlighted in grey. Data was extracted from condition-dependent transcriptome by Nicolas *et al*. 2012 (13).

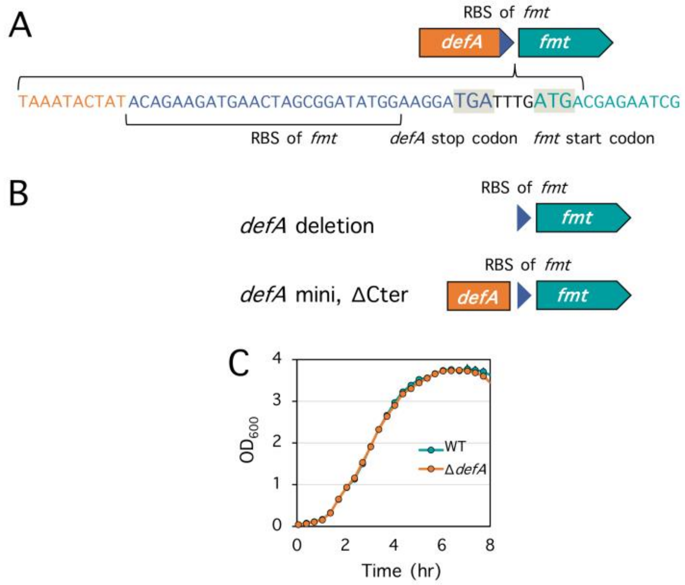

Fig. S2. Construction of *defA* mutants. (A) Schematic representation of the modular architecture of *defA-fmt* operon. The RBS (ribosomal binding site) of *fmt* is overlapped with *defA* ORF. (B) Schematic representation of *defA* mutants construction. To enable the proper expression of *fmt*, the RBS of *fmt* was maintained as integrity during the genetic construction. (C) Growth curve of *B. subtilis* wild type and Δ*defA* strains. Growth was measurement in BioLector ® I high-throughput microbioreactor with flower 48-well plates. Data are presented as mean ± SD.

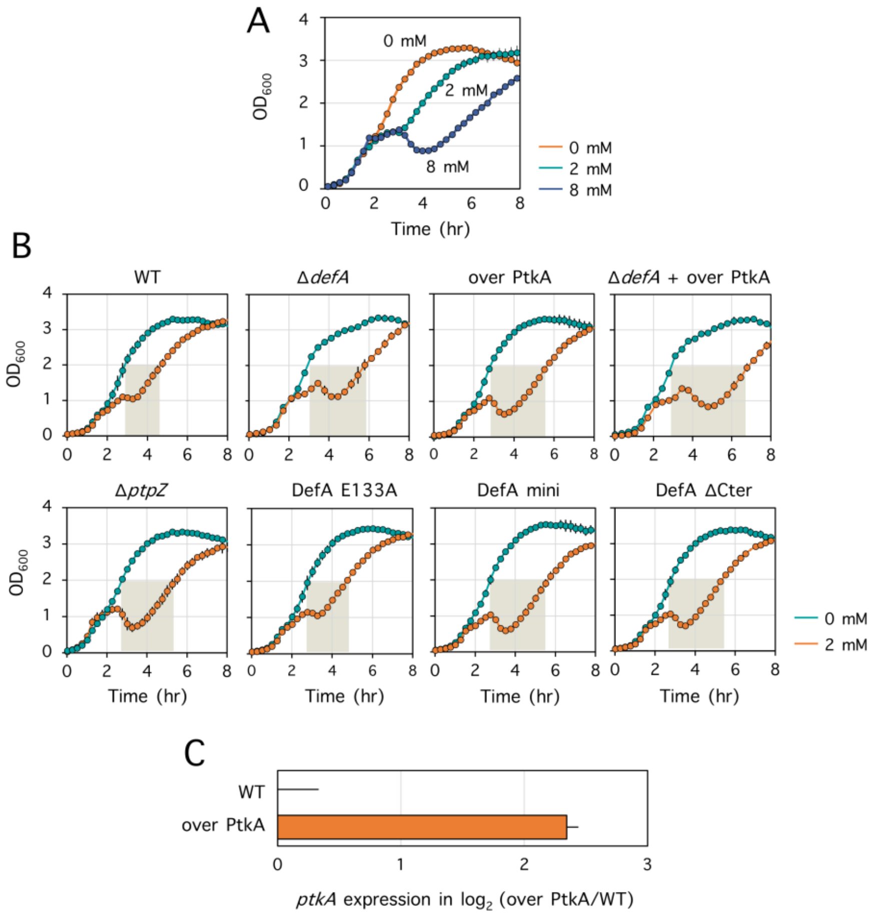

Fig. S3. Growth phenotype of *B. subtilis* wild type and relevant mutants upon oxidative stress. (A) Growth curve of *B. subtilis* wild type with H_2O_2 concentration of 0, 2 and 8 mM. Growth was measured in BioLector ® I high-throughput microbioreactor with flower 48-well plates. H_2O_2 was added into culture medium when OD600 reaches 0.6. Data are presented as mean ± SD. (B) Growth curve of *B. subtilis* strains with H_2O_2 treatment, measured in BioLector ® I with flower 48-well plates. 2 mM H_2O_2 was added into culture medium at OD_{600} 0.6. Data are presented as mean ± SD. The shaded rectangle crossed on the y-axis indicates the TR (time of recovery, Fig. 1D). (C) Expression of PtkA in over PtkA strain. Expression was measurement by quantitative reverse-transcriptional PCR (qPCR). PtkA expression in wild type was normalized as 1. The change is expressed as log_2 ratio. Data are presented as mean + SD.

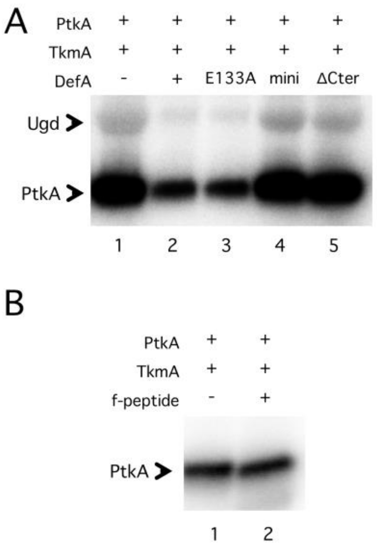

Fig. S4. *In vitro* phosphorylation assay of Ugd and PtkA. (A) *In vitro* phosphorylation of Ugd by PtkA in the presence of DefA. The presence or absence of proteins is indicated as ± above each lane. Phosphorylation signals were revealed by autoradiography. Bands corresponding to phosphorylation of Ugd and PtkA are indicated by arrows. (B) *In vitro* autophosphorylation PtkA in absence and presence of f-peptide.

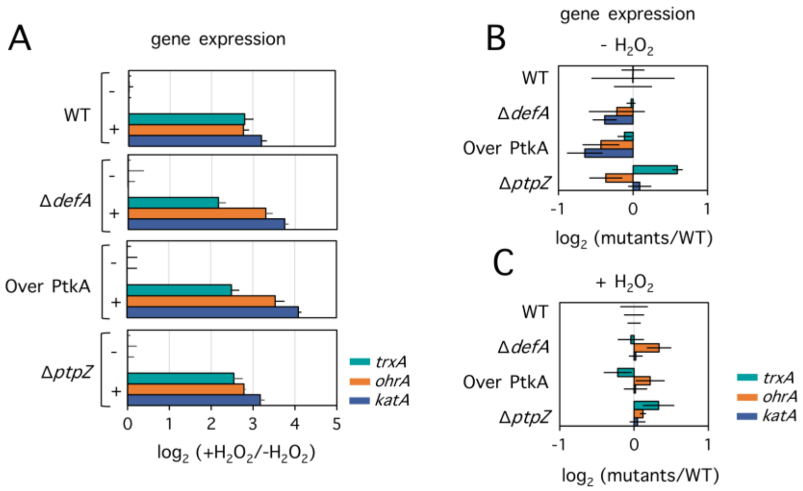

Fig. S5. Expression of marker genes for PerR, OhrR and Spx regulons (katA, ohrA, and trxA) in wild type, Δ*defA*, Over PtkA and Δ*ptpZ*. (A) Expression without H_2O_2 treatment in mutants versus in wild type. The expression in wild type was normalized as 1. Data are presented as mean ± SD. (B) Expression with H_2O_2 treatment in mutants versus in wild type. The expression in wild type was normalized as 1. Data are presented as mean ± SD.

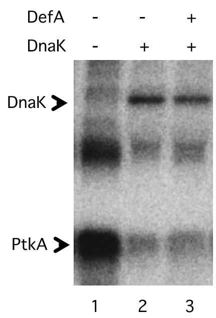

Fig. S6. *In vitro* phosphorylation of DnaK by PtkA in presence of DefA. The presence or absence of proteins is indicated as ± above each lane. Phosphorylation signals were revealed by autoradiography. Bands corresponding to phosphorylation of DnaK and PtkA are indicated by arrows.

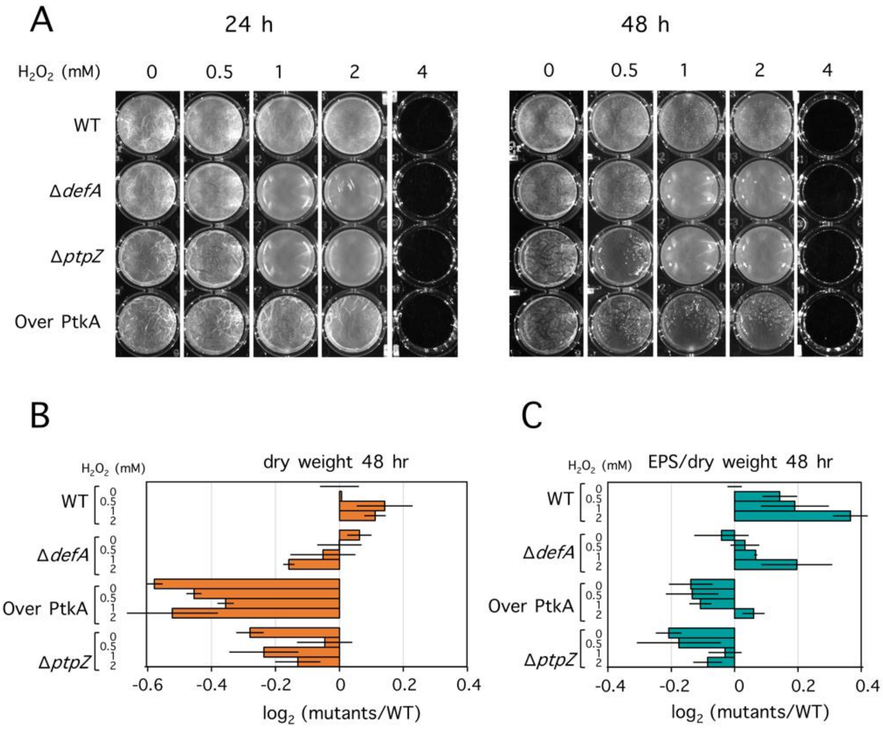

Fig. S7. Pellicle formation and quantification of *B. subtilis* wild type, Δ*defA,* Over PtkA and Δ*ptpZ.* (A) Representative image of pellicle formation of *B. subtilis* wild type, Δ*defA, ΔptpZ* and Over PtkA treated with different concentrations of H_2O_2: 0, 0.5, 1, 2 and 4 mM. Cells were inoculated in the MSgg medium and grown for 24 and 48 hours at 37°C to allow pellicle formation. (B) Measurement of dry weight per pellicle of wild type, Δ*defA*, Over PtkA and Δ*ptpZ*. Pellicles were formed in the presence of H_2O_2 with different concentration (0, 0.5, 1 and 2 mM) in Msgg medium for 48 hr. The dry weight of wild type at 0 mM H_2O_2 was normalized as 1. (C) Measurement of EPS per dry weight of wild type, Δ*defA*, Over PtkA and Δ*ptpZ*.

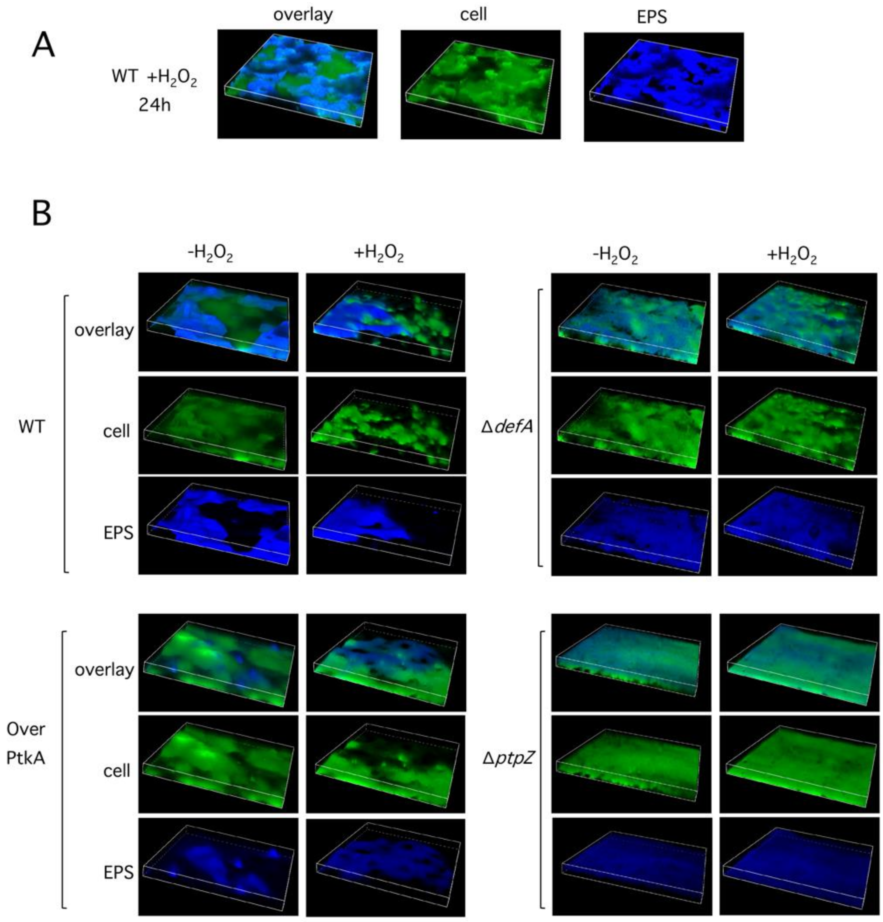

Fig. S8. EPS distribution pattern in pellicles developed by wild type, Δ*defA*, Over PtkA and Δ*ptpZ* in absence and presence of H_2O_2. For each strain, blue fluorescence indicates EPS stained with Alexa Fluor 594 Conjugated Concanavalin A (right panel), green fluorescence indicates cells stained with SYTO 9 nucleic acid stain (middle panel), and they are also shown in overlay (left panel). The Z-axis range was 48.75 μm. (A) Pellicles developed by wild type at 24 h. Z-axis was moved down to the layer of cells. (B) Pellicles developed at 48 h.

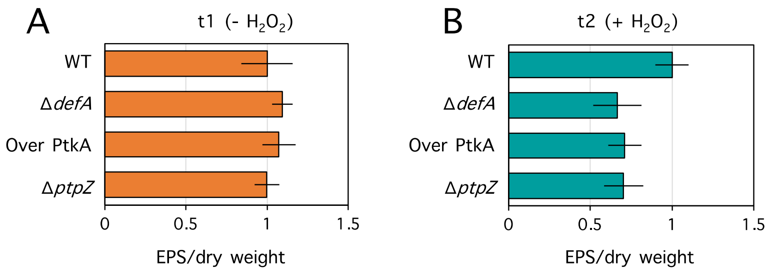

Fig. S9. Measurement of EPS/dry weight of wild type, Δ*defA*, Over PtkA and Δ*ptpZ* in planktonic form. Cells were cultured in LB medium. Samples were taken at (A) t1 (indicated in Fig. 1C) in absence of H_2O_2 and (B) t2 (indicated in Fig. 1C) in presence of H_2O_2. EPS/dry weight of wild type was normalized as 1.

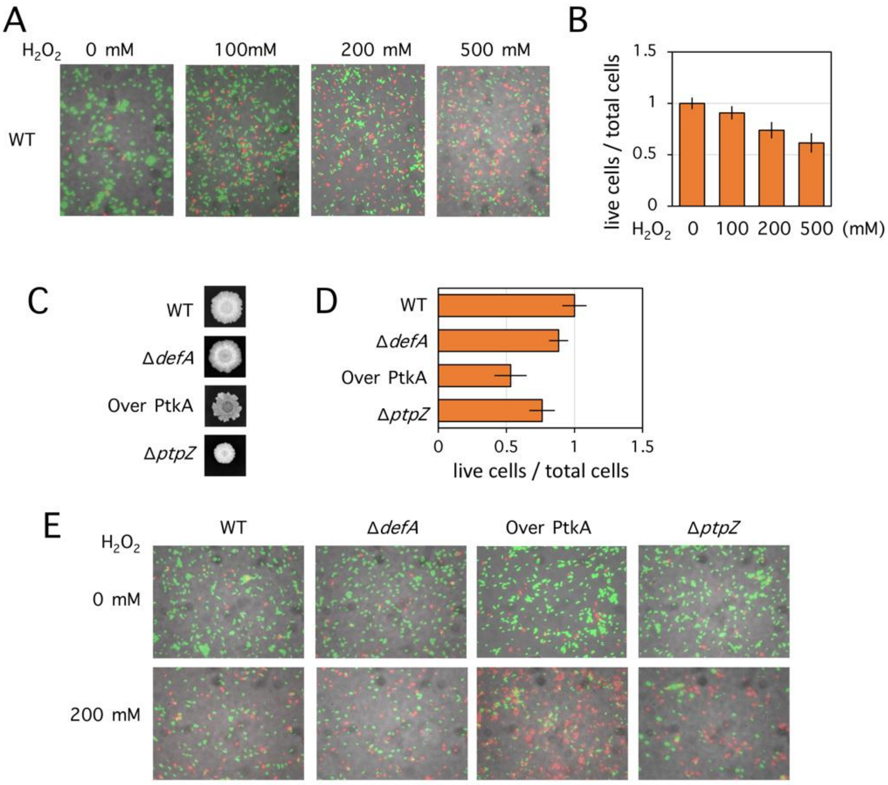

Fig. S10. Resistance of pre-established biofilms to H_2O_2 treatment. Complex colonies were developed on Msgg plates and exposed to H_2O_2 for 2 h. Live/dead cells were visualized by LIVE/DEAD BacLight Bacterial Viability staining. (A) Representative microscope images of wild type treated with 0, 100, 200 and 500 mM H_2O_2. Red fluorescence indicates dead cells stained with propidium iodide, green fluorescence indicates live cells stained with SYTO 9 nucleic acid stain. The overlay of white field, red fluorescence and green fluorescence is presented. (B) Survival of wild type treated with different concentration of H_2O_2. The ratio of live cells to total cells is presented. The live cells were indicated by green fluorescence and total cells were a sum of cells with green fluorescence and those with red fluorescence. Wild type without treatment was normalized as 1. (C) Represented image of complex colony of wild type, Δ*defA*, Over PtkA and Δ*ptpZ* in Msgg plate. (D) Survival of wild type, Δ*defA*, Over PtkA and Δ*ptpZ* in H_2O_2 treatment. (E) Represented images of wild type, Δ*defA*, Over PtkA and Δ*ptpZ* in H_2O_2 treatment.

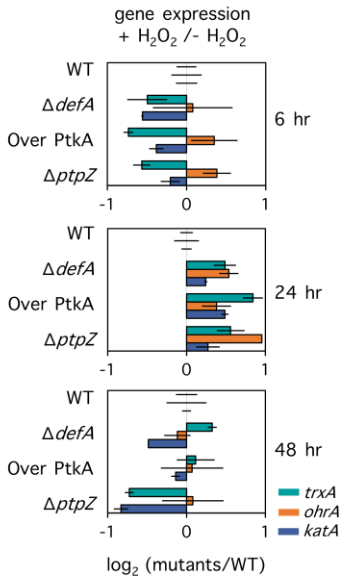

Fig. S11. Expression of marker genes for PerR, OhrR and Spx regulons. Expression of *katA*, *ohrA*, and *trxA* in wild type, Δ*defA*, Over PtkA and Δ*ptpZ* during pellicle formation in Msgg medium at 6, 24 and 48 hr. The ratio of gene expression in wild type with H_2O_2 to that without H_2O_2 was normalized as 1. Data are presented as mean ± SD.

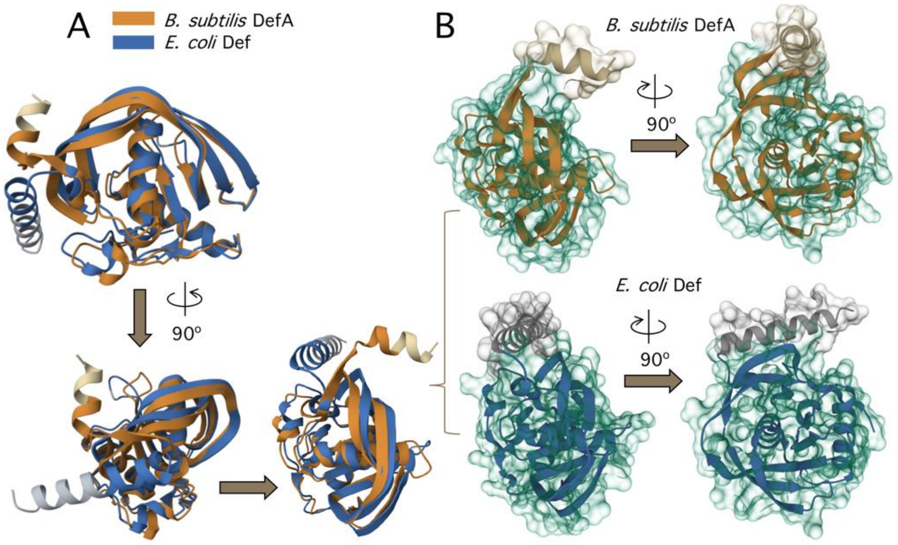

Fig. S12. Protein structure of *B. subtilis* DefA and *E. coli* Def. (A) Structure alignment of *B. subtilis* DefA and *E. coli* Def. The only part of the structure that does not align, the C-terminal α-helix, is highlighted in gold for *B. subtilis* DefA and gray for *E. coli* Def. (B) Molecular surface of the *B. subtilis* DefA and *E. coli* Def. Both PDFs are shown as ribbons diagram in the same orientation. Images of protein structures were created by Mol* (9, 10).

Supplemental Table

Supplementary Table S1: Oligos used in this study.

name	sequences (5'-3')
**pMAD_defA133_up_fd**	GCGAGAGTGATTCGGATGGATGTTGA
**pMAD_defA133_up_rs**	ACAGCACACCGTCTAAGTGGTCCATTGCATGCTGCACGGCTCTTG CTAA
**pMAD_defA133_dw_fd**	TTAGCAAGAGCCGTGCAGCATGCAATGGACCACTTAGACGGTGT GCTGT
**pMAD_defA133_dw_rs**	GTAATGTACCATAGACAAGTTCAGTCA
**pMAD_defA133_fd_eco**	CCGGAATTCAACACTTCATATTCCTGATTTCAGAT
**pMAD_defA133_rs_bam**	CGCGGATCCCATTGTTTACTCCTAACACATCACC
**physpank_fd_new**	CCGGAATTCCTCGAGGGTAAATGTGAGCACTCAC
**physpank_rs**	CTAGACTAGTGGGCCCAGATCTCATCCTAGGAATCTCCTTTCTAA TTGTTATCCGCTCACAATTACACA
**physpank_ptkA_fd**	GGAAGATCTATGGCGCTTAGAAAAAACAGAGGCTC
**physpank_ptkA_rs**	AAAACTGCAGTTATTTTTGCATGAAATTGTCCTTGGTTC
**pMAD_ptpZ_up_fd**	CGCGGATCCTCCCAAGTGGTCAACATATCAGTCAG
**pMAD_ptpZ_up_rs**	GTGGTGAAATGAGAATCGGCTGTTACATGTCCTAGCCCCCTTTTT TCGCAA
**pMAD_ptpZ_dw_fd**	TTGCGAAAAAAGGGGGCTAGGACATGTAACAGCCGATTCTCATTT CACCAC
**pMAD_ptpZ_dw_rs**	ACGCGTCGACCTAACTGCAATGGGATCGTAGGCTC
**pMAD_defA_fd**	GGCTGAGCATCACAGCTGCCCGGTTG
**pMAD_defA_rs**	GTAATGTACCATAGACAAGTTCAGTCA
**pMAD_defA_up_rs**	CCATATCCGCTAGTTGATCTTCTGTTTTGTTACCCTCCAAGACGTG AGATC
**pMAD_defA_dw_fd**	GATCTCACGTCTTGGAGGGTAACAAAACAGAAGATCAACTAGCGG ATATGG
**pMAD_defA_fd_eco**	CCGGAATTCGAGAGAGGAGCTTAGAAACGGCAATCG
**pMAD_defA_rs_bam**	CGCGGATCCCATTGTTTACTCCTAACACATCACC
**pMAD_pm_defA_fd**	GCGAGAGTGATTCGGATGGATGTTG
**pMAD_pm_defA_rs**	TGTTCTAATTTGATCAGCGCCTCAAG
**pMAD_pm_defA_fd_eco**	CCGGAATTCAACACTTCATATTCCTGATTTCAGATC
**pMAD_pm_defA_rs_bam**	CGCGGATCCGTTCGGTTAATAACAGAGCCGTCTCG
**pMAD_pm_def-deltaC_up_rs**	CCATATCCGCTAGTTGATCTTCTGTTCATAGTTCATCTTCTGTATA GTA
**pMAD_pm_def-deltaC_dw_fd**	TACTATACAGAAGATGAACTATGAACAGAAGATCAACTAGCGGAT ATGG
**pMAD_defA-mini_fd_eco**	CCGGAATTCTTCCTTTGTGATGTGCAGGGATTGC

**pMAD_defA-mini_rs_bam**	CGCGGATCCGTTCGGTTAATAACAGAGCCGTCTCG
**pMAD_defA-mini_fd**	GAGAGAGGAGCTTAGAAACGGCAATCG
**pMAD_defA-mini_rs**	TGTTCTAATTTGATCAGCGCCTCAAG
**pMAD_defA-mini_up_rs**	CTGTCATCCCCGATTTCTACGACGGCTTTGTTACCCTCCAAGACG TGAGAT
**pMAD_defA-mini_mid_fd**	ATCTCACGTCTTGGAGGGTAACAAAGCCGTCGTAGAAATCGGGG ATGACAG
**pMAD_defA-mini_mid_rs**	TCCATATCCGCTAGTTGATCTTCTGTTCATAGTTCATCTTCTGTATA GTATT
**pMAD_defA-mini_dw_fd**	AATACTATACAGAAGATGAACTATGAACAGAAGATCAACTAGCGG ATATGGA
**pQE_defA_deltaC_fd**	CGCGGATCCATGGCAGTAAAAAAGGTCGTCACACATCC
**pQE_defA_mini_fd**	CGCGGATCCATGGCCGTCGTAGAAATCGGGGATGA
**pQE_defA_deltaC/min_rs**	AAAACTGCAGTCATAGTTCATCTTCTGTATAGTATTTAC
**pQE_defB_fd**	CGCGGATCCATGATTACTATGGAAAACATCG
**pQE_defB_rs**	AAAACTGCAGTTAGCGCTCAATTGCGATTGC
**pQE_defA_fd**	CGCGGATCCATGGCAGTAAAAAAGGTCG
**pQE_defA_rs**	AAAACTGCAGTTATCCTTCCATATCCGCTAGTTC
**pQE_defA133_fd**	GCAGCATAAGATGGACCACTTAGACGGTGTG
**pQE_defA133_rs**	GGTCCATCTTATGCTGCACGGCTCTTGC
**pQE_defA95_fd**	CTTTCCTACCGTCTATGGTGATGTCACACGTG
**pQE_defA95_rs**	CATAGACGGTAGGAAAGCTCAAGCATCCTTC
**pQE_defA150_rs**	AAAACTGCAGTTATCCTTCCATATCCGCTAGTTCATCTTCTGTAGC GTATTTACTTA
**pGAD-C1_defA_fd**	CCGGAATTCATGGCCGTCGTAGAAATCGGGGATG
**pGAD-C1_defA_rs**	CGCGGATCCTCATCCTTCCATATCCGCTAGTTC
**qPCR_rpsU_F**	AAGATGCTCTTCGTCGCTTC
**qPCR_rpsU_R**	TGCGTTTTCTAGCAGCTTCA
**qPCR_ohrA_F**	CAGCTGTTTGCAGCAGGTTA
**qPCR_ohrA_R**	GCCTCCAATTCTGATGCACT
**qPCR_katA_F**	ATCATGATGCACACCGCTAC
**qPCR_katA_R**	CCGAAGCTGTTAGGCTCGTA
**qPCR_trxA_F**	AGACTTCTGGGCTCCTTGGT
**qPCR_trxA_R**	AAGAAGAGTCGGGATGCTCA

Supplemental references

1. L. Shi *et al.*, Protein-tyrosine phosphorylation interaction network in *Bacillus subtilis* reveals new substrates, kinase activators and kinase cross-talk. *Front Microbiol* **5**, 538 (2014).
2. M. F. Noirot-Gros *et al.*, An expanded view of bacterial DNA replication. *P Natl Acad Sci USA* **99**, 8342-8347 (2002).
3. I. Mijakovic et al., Transmembrane modulator-dependent bacterial tyrosine kinase activates UDP-glucose dehydrogenases. Embo J 22, 4709-4718 (2003).
4. S. Kumar et al., Identification of crucial amino acids of bacterial peptide deformylases affecting enzymatic activity in response to oxidative stress. J Bacteriol 196, 90-99 (2014).
5. A. Anjem A, J. A. Imlay, Mononuclear iron enzymes are primary targets of hydrogen peroxide stress. J Biol Chem 287, 15544-56 (2012).
6. X. Che et al., Expression, purification, and activity assay of peptide deformylase from *Escherichia coli* and *Staphylococcus aureus*. Mol Cell Biochem 357, 47-54 (2011).
7. P. B. Beauregard et al., *Bacillus subtilis* biofilm induction by plant polysaccharides. Proc Natl Acad Sci U S A 110, E1621-30 (2013).
8. S. Pandit et al., Effect of sodium fluoride on the virulence factors and composition of *Streptococcus* mutans biofilms. Arch Oral Biol 56, 643-9 (2011).
9. N. Peng et al., The exopolysaccharide–eDNA interaction modulates 3D architecture of *Bacillus subtilis* biofilm. BMC Microbiol 20, 1–12 (2020).
10. S. Pandit et al., Low concentrations of vitamin C reduce the synthesis of extracellular polymers and destabilize bacterial biofilms. Front Microbiol 8, 320391 (2017).
11. T. Bucher et al., Methodologies for studying *B. subtilis* biofilms as a model for characterizing small molecule biofilm inhibitors. J Vis Exp 9, 54612 (2016).
12. C. García et al., Metal ions weaken the hydrophobicity and antibiotic resistance of *Bacillus subtilis* NCIB 3610 biofilms. NPJ Biofilms Microbiomes 3, 1 (2020).
13. J. Jumper et al., Highly accurate protein structure prediction with AlphaFold. Nature 596, 583-589 (2021).
14. D. Kozakov et al., The ClusPro web server for protein-protein docking. Nat Protoc 12, 255-278 (2017).
15. V. Olivares-Illana et al., Structural basis for the regulation mechanism of the tyrosine kinase CapB from *Staphylococcus aureus*. Plos Biol 6, 1321-1331 (2008).
16. E. Krieger et al., Improving physical realism, stereochemistry, and side-chain accuracy in homology modeling: Four approaches that performed well in CASP8. Proteins 77, 114-122 (2009).
17. H. M. Berman et al., The Protein Data Bank. Nucleic Acids Res 28, 235-242 (2000).
18. D. Sehnal et al., Mol* Viewer: modern web app for 3D visualization and analysis of large biomolecular structures. Nucleic Acids Res 49, W431-W437 (2021).
